# In Silico Approach for Early Antimalarial Drug Discovery: De Novo Design of Virtual Multi-Strain Antiplasmodial Inhibitors

**DOI:** 10.3390/microorganisms13071620

**Published:** 2025-07-09

**Authors:** Valeria V. Kleandrova, M. Natália D. S. Cordeiro, Alejandro Speck-Planche

**Affiliations:** LAQV@REQUIMTE/Department of Chemistry and Biochemistry, Faculty of Sciences, University of Porto, 4169-007 Porto, Portugal; valeria.kleandrova@gmail.com (V.V.K.); ncordeir@fc.up.pt (M.N.D.S.C.)

**Keywords:** PTML, topological indices, multilayer perceptron, fragment, fragment-based topological design, malaria

## Abstract

*Plasmodium falciparum* is the causative agent of malaria, a parasitic disease that affects millions of people in terms of prevalence and is associated with hundreds of thousands of deaths. Current antimalarial medications, in addition to exhibiting moderate to serious adverse reactions, are not efficacious enough due to factors such as drug resistance. In silico approaches can speed up the discovery and design of new molecules with wide-spectrum antimalarial activity. Here, we report a unified computational methodology combining a perturbation theory machine learning model based on multilayer perceptron networks (PTML-MLP) and the fragment-based topological design (FBTD) approach for the prediction and design of novel molecules virtually exhibiting versatile antiplasmodial activity against diverse *P. falciparum* strains. Our PTML-MLP achieved an accuracy higher than 85%. We applied the FBTD approach to physicochemically and structurally interpret the PTML-MLP, subsequently extracting several suitable molecular fragments and designing new drug-like molecules. These designed molecules were predicted as multi-strain antiplasmodial inhibitors, thus representing promising chemical entities for future synthesis and biological experimentation. The present work confirms the potential of combining PTML modeling and FBTD for early antimalarial drug discovery while opening new horizons for extended computational applications for antimicrobial research and beyond.

## 1. Introduction

Malaria constitutes an incredibly serious medical condition characterized by a deadly impact on the human population. This mosquito-borne infectious disease was responsible for about 247 million malaria cases and 619,000 malaria deaths [1]. Among the causative agents of malaria, *Plasmodium falciparum* accounts for the majority of incidence and mortality cases, representing the most concerning parasitic pathogen [2,3]. In addition, current antimalarial drugs have become less effective due to the development of and increase in drug resistance, including multidrug-resistant (MDR) strains [4,5,6]. All of these factors, together with the adverse effects associated with current antimalarial medications [7,8], indicate that the search for new antiplasmodial agents against *P. falciparum* continues to be a highly active field of research, as well as a need to tackle malaria.

Over time, antimalarial drug discovery has evolved to accelerate the identification of a variety of new chemical entities, with some of them being in late-stage clinical development [1,9,10]. Although experimental methods constitute the benchmark to deliver and validate antiplasmodial agents against *P. falciparum* malaria [11], they are associated with a great expenditure of time and financial resources. In this sense, discovering antiplasmodial agents against *P. falciparum* can be rationalized through in silico approaches, with complex network analysis [12], pharmacophore modeling [12,13,14], quantum mechanical calculations [13,15,16], structure-based drug design methods (e.g., molecular docking alone or in combination with molecular dynamics) [12,13,14,15,16,17,18,19,20], and machine learning [20,21,22]. Nevertheless, one or more limitations, such as the use of reduced dataset of chemicals (impeding an appropriate prioritization of the vast chemical space), the prediction of activity against only one protein or strain related to *P. falciparum* (thus affecting the search for antiplasmodial molecules capable of simultaneously targeting different strains), and insufficient physicochemical or structural interpretability (preventing the rational design of novel chemical entities with multi-strain antiplasmodial activity), are present in the aforementioned computational approaches, preventing their full exploitation for the discovery of antiplasmodial compounds against *P. falciparum*.

Advanced models based on perturbation theory machine learning (PTML) have been able to overcome the disadvantages mentioned above [23,24]. In terms of applications, PTML models have been successfully employed in antimicrobial research [25,26,27,28,29,30], neurological diseases [31,32,33,34,35], immunology [36,37,38], nanocarriers [39,40,41], and antineoplastic discovery [39,42,43,44,45,46]. Furthermore, PTML models have been directly interpreted by applying the fragment-based topological design (FBTD) approach, thus enabling the de novo design of molecules virtually exhibiting the desired bioactivity profiles [30,46].

Despite the pivotal role of all the aforementioned in silico methods in prioritizing drug discovery, there have been no studies focused on guiding the de novo rational design of multi-strain antiplasmodial inhibitors at the phenotypic level. Here, we set the theoretical bases for the applications of PTML modeling to early-stage antimalarial discovery. In particular, we demonstrate that the computational framework combining a PTML model based on a multiplayer perceptron network (PTML-MLP) and the FBTD approach can be used to enable the interpretation-driven de novo design of new drug-like molecules with predicted multi-strain antiplasmodial activity to be considered for future synthesis and biological experimentation.

## 2. Materials and Methods

### 2.1. Data Curation and Topological Indices

**Figure 1:**
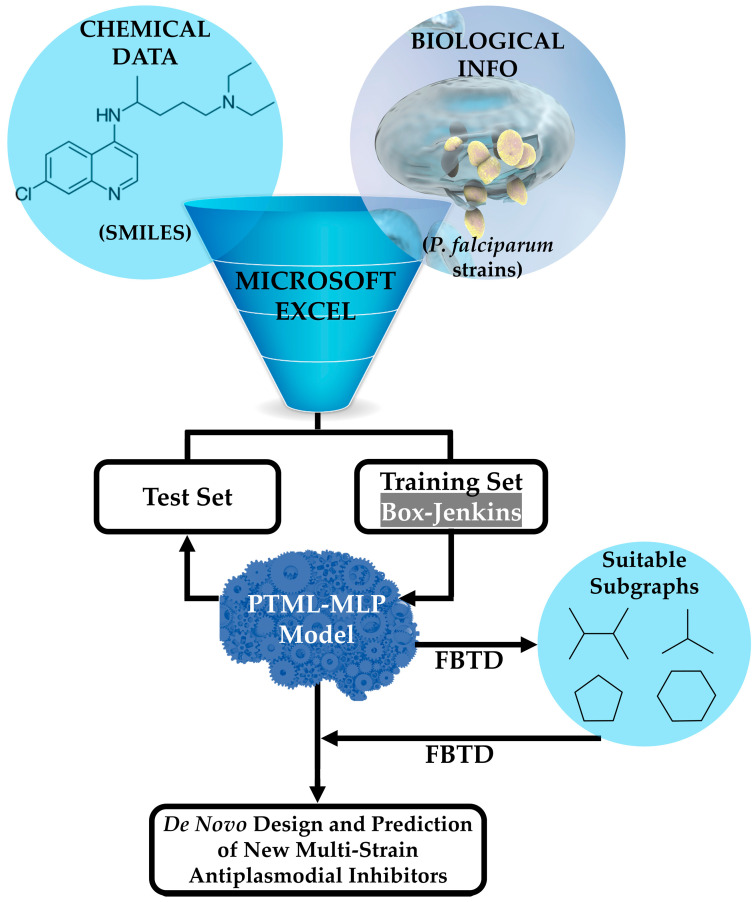
Combination of a PTML-MLP and the FBTD approach to enable the prediction and de novo design of versatile antiplasmodial chemicals. The training and test sets are used to find the best PTML-MLP and assess the predictive power, respectively. Microsoft Excel, as a math editor and tabulator, enables the use of different mathematical formulas related to the Box–Jenkins approach (see Equations (1) and (2)). The FBTD approach is applied to perform two tasks, namely the physicochemical and substructural interpretation of the PTML-MLP with the subsequent extraction of the subgraphs (in the form of molecular fragments such as functional groups or rings) responsible for the multi-strain antiplasmodial activity and the fusion and connection of different suitable fragments, yielding new drug-like molecules virtually exhibiting multi-strain antiplasmodial activity. More details on the application of the FBTD approach will be given in Section 3.

The general steps associated with the joint use of the PTML-MLP and the FBTD approach are illustrated in Figure 1. Details will be given throughout this entire Materials and Methods section, as well as some key aspects regarding the application of FBTD in Section 3. In this sense, a single tabular file compatible with Microsoft Excel was downloaded from the public online database known as ChEMBL [47,48,49]. The file contained both the chemical data in the form of Simplified Molecular Input Line Entry System (SMILES) codes and the biological information, represented by the half-maximal inhibitory concentration (IC_50_) expressed in nanomolar (nM) against *P. falciparum*. The IC_50_ values considered in this study were measured over a time of either 48 or 72 h, since a recent report demonstrated that the IC_50_ values do not show a significant difference when measured by considering the aforementioned periods [50,51]. Consequently, as part of the curation process (containing steps such as deleting entries lacking SMILES codes, units of measurement, and IC_50_ values), when a molecule was assayed more than once against the same *P. falciparum* strain, we kept only the entry corresponding to the lowest IC_50_ value of that molecule. It is important to highlight that after the curation process, the dataset contained 6513 molecules, where each of them was experimentally assayed against at least 1 out of 9 *P. falciparum* strains (*tg*). Not all of the molecules were tested against all the *P. falciparum* strains (*tg*), and thus the dataset reported in this study ended up containing 9595 cases.

Each case of molecules in the dataset was classified as active or inactive. If a molecule had IC_50_ ≤ 500 nM against a specific *P. falciparum* strain, then the molecule was labeled as active [*APi*(*ej*) = 1], while molecules with IC_50_ ≥ 2000 nM were noted as inactive [*APi*(*ej*) = –1]. The selection of these cutoffs prevented any imbalance between the number of active and inactive cases, while the non-inclusion of molecules with intermediate activity (500 nM < IC_50_ < 2000 nM) guaranteed a sufficiently clear demarcation between the aforementioned activity classes [52]. Notice that *APi*(*ej*) is a two-category variable accounting for the antiplasmodial activity of the *i*th case under the experimental condition *ej*. At the same time, *ej* contains two aspects, namely *tg* (containing 9 labels, each of them belonging to a specific type of *P. falciparum* strain) and *ds*, with the latter containing two labels indicating whether a defined *P. falciparum* strain was sensitive or resistant to well-established antimalarial drugs (chloroquine, quinine, pyrimethamine, sulfadoxine, and cycloguanil).

The SMILES codes of the 9595 cases of molecules were deposited in a .txt file. This file was used as input by the software MODESLAB version 1.5 [53], where the topological indices (*TIs*) known as bond-based spectral moments [*SM*(*PP*)*o*], degree-based connectivity indices from the vertices’ valences [*Xv*(*SG*)*m*] and edges [*e*(*SG*)*m*], and the Kier–Hall shape index [*K*(*Alpha*)*m*] were calculated [54,55]. Notice that in the case of *SM*(*PP*)*o*, the notation “*o*” indicated the order (maximum number of bonds that a fragment can have, with *o* being between 1 and 7), while “*PP*” referred to any physicochemical property, such as the standard bond distance and bond dipole moment, or atomic contributions to hydrophobicity, the polar surface area, molar refractivity, Gasteiger-Marsili charges, and atomic weight. Regarding *Xv*(*SG*)*m* and *e*(*SG*)*m*, the order “*m*” reflects the specific size (exact number of bonds, with *m* in the range from 1 to 6) of a subgraph or fragment *SG*. Furthermore, the fragment *SG* was present in four types, namely path (linear fragment), cluster (ramification), path-cluster (combination of linear portion and ramification), and cycle (ring). Lastly, for the case of *K*(*Alpha*)*m*, this considers *m* = 3 for only path subgraphs, while “*Alpha*” is a factor considering the presence of heteroatoms (non-hydrogen atoms other than carbon) [54,55]. We also calculated a new set of normalization-like topological indices (*NTIs*). Each *NTI* was obtained as the ratio of a *TI* to *nBO*, with the latter representing the number of bonds in a molecule without counting bond multiplicity.

The dataset containing the 9595 cases of molecules (Supplementary Information S1) was divided into training (~75%) and test (~25%) sets according to the following procedure. First, the 9595 cases were ordered in terms of their increasing IC_50_ values. Then, the first three cases were annotated to belong to the training set, while the fourth was designated to be in the test set. The procedure was repeated until all the cases were assigned to the training or the test sets. After assigning all the cases to the training and test sets, we applied the Box–Jenkins approach through the following mathematical steps [30,46,56]:

(1) $$avg\left[GBI\right]ej=\frac{1}{n\left(ej\right)}\times \overset{n\left(ej\right)}{\underset{a=1}{\sum }}GBI_{a}$$

(2) $$D\left[GBI\right]ej=\left[\frac{GBI-avg\left[GBI\right]ej}{sdv\left[GBI\right]}\right]\times \sqrt{p\left(ej\right)}$$

In Equation (1), *GBI* represents any of the original *TIs* (or their normalized counterparts (*NTIs*)) calculated for each case or molecule in the dataset. The term *n*(*ej*) indicates the number of cases annotated as active, which were experimentally tested against the same element of *ej*. Because *ej* depends on the aspects *tg* and *ds*, Equation (1) was applied to each of them separately. The same assumption was valid for the average term *avg*[*GBI*]*ej*. On the other hand, Equation (2) was also applied to the aspects *tg* and *ds* separately. Thus, the a priori probability *p*(*ej*) followed the same assumption as *n*(*ej*) and *avg*[*GBI*]*ej*, being calculated as the ratio of *n*(*ej*) to the total number of cases tested against the same aspect of *ej* (*tg* or *ds*). Furthermore, *sdv*[*GBI*] reflected the standard deviation of all the *GBI* values. The *D*[*GBI*]*ej* term is a multi-label graph index accounting for both the chemical structure of a case or molecule and a specific aspect of the experimental condition *ej* (*tg* or *ds*). Notice that *n*(*ej*), *avg*[*GBI*]*ej*, and *p*(*ej*) were calculated from cases of molecules labeled as belonging to the training set.

### 2.2. PTML Modeling: Assessment of Performance and Applicability Domain

The next step after calculating the *D*[*GBI*]*ej* indices was to rank them in terms of significance or information content. For this, we employed the computer program IMMAN version 1.0 [57]. In this sense, for each *D*[*GBI*]*ej* index, we calculated the geometric mean value (*GMV*) of three information-based metrics, namely the differential Shannon entropy [58], gain ratio [59], and symmetric uncertainty [60]. The *D*[*GBI*]*ej* indices with the greatest information content (and potentially the greatest discriminatory power) were those exhibiting the largest *GMVs*. Following the sorting of the *D*[*GBI*]*ej* indices according to their decreasing *GMVs*, we performed a correlation analysis, calculating the pair-wise Pearson’s correlation coefficient (*PCC*) values. The software STATISTICA version 13.5.0.17 was employed to perform the correlation analysis [61]. We only chose those *D*[*GBI*]*ej* indices satisfying the condition −0.7 < *PCC* < 0.7; the other *D*[*GBI*]*ej* indices were discarded.

The last step was to find the PTML-MLP, using the *D*[*GBI*]*ej* indices as its inputs. Through this procedure, we employed the artificial neural network menu of the software STATISTICA version 13.5.0.17. When searching for the most appropriate MLP network (PTML-MLP), we configured the values of the key hyperparameters. Thus, the number of input nodes (*N_i_*) was set to be 25, and the minimum and maximum numbers of hidden neurons (*H_n_*) were 30 and 80, respectively. The logistic and hyperbolic tangents were used as the activation functions in both the hidden and output layers. The number of output nodes (*O_n_*) was set to 2 (number of predicted categories, i.e., active and inactive).

We configured the procedure to train 1000 MLP networks, with 300 of them being retained. The values of the different aforementioned hyperparameters selected by us were based on our extensive experience in PTML modeling, which allowed us to compare the dataset used in the present study with the datasets reported in previous PTML-MLPs [30,46,56]. Thus, we could estimate the hyperparameters mentioned above. The parameter *ρ* was calculated to assess whether the MLP networks could lead to overfitting:

(3) $$\rho =\frac{C_{t}}{\left[\left(N_{i}+1\right)H_{n}+(H_{n}+1\right)O_{n}]}$$

In Equation (3), the meanings of *N_i_*, *H_n_*, and *O_n_* were explained above. The number of cases in the training set (*C_t_*) is also considered in this equation. To avoid overfitting, the condition *ρ* > 3 must be complied with [62,63]. In the end, we checked the performance of 300 retained MLP networks utilizing the global metrics (in both the training and test sets) known as sensitivity (*Sn*), specificity (*Sp*), and the normalized Matthew’s correlation coefficient (*nMCC*) [64]. However, the most suitable MLP network (the PTML-MLP) was the one exhibiting the highest values of local sensitivities [*Sn*(*tg*) and *Sn*(*ds*)] and specificities [*Sp*(*tg*) and *Sp*(*ds*)]. These local metrics depended on the two aspects of the experimental condition *ej* (*tg* and *ds*). Last, the applicability domain (AD) of the PTML-MLP was assessed according to a recent variation of the descriptors’ space approach [30,46,56].

## 3. Results and Discussion

### 3.1. Analyzing the Performance of the PTML-MLP Model

Details on the *D*[*GBI*]*ej* indices used as inputs by the PTML-MLP are present in Table 1. These include definitions based on the physicochemical and structural aspects.

The PTML-MLP found by us can be expressed in the form MLP 25-78-2. This notation means that the number of input nodes is *N_i_* = 25 (equal to the number of *D*[*GBI*]*ej* indices), the number of neurons in the hidden layer is *H_n_* = 78, the number of output nodes is *O_n_* = 2, and the number of cases is *C_t_* = 7197 (Supplementary Information S1). Substitution of the *N_i_*, *H_n_*, *O_n_*, and *C_t_* values into Equation (3) yielded a value of *ρ* = 3.292. Such a value confirms that our PTML-MLP did not overfit the data.

We analyzed the performance of the PTML-MLP at the global and local levels. In terms of global metrics (Table 2), the PTML-MLP exhibited *Sn* values above 93% and 89% in the training and test sets, respectively. This indicates a high number of true positive (*TP*) cases relative to the number of cases annotated as active (*N*_Active_). A similar trend was observed in the *Sp* values, where the number of true negative (*TN*) cases relative to the number of cases labeled as inactive (*N*_Inactive_) was also high. For the training set, *Sp* > 90% was obtained, while *Sp* > 86% was achieved in the test set.

The *Sn* and *Sp* values indicate that our PTML-MLP has great statistical quality and predictive power when classifying antiplasmodial chemicals. This affirmation is further corroborated by the high *nMCC* values; the closeness of these *nMCC* values to one shows the strong convergence between the observed *APi*(*ej*) and predicted *Pred*[*APi*(*ej*)] values of antiplasmodial activity. More information regarding the classification and predictions performed by the PTML-MLP can be found in the Supplementary Information S2 File. Aside from achieving extremely good values for *Sn*, *Sp*, and *nMCC*, our PTML-MLP displayed quite good values for the local metrics. In this sense, *Sn*(*tg*) was in the interval 88–98% in the training set; in the test set, *Sn*(*tg*) was in the range of 80–93%. At the same time, *Sp*(*tg*) exhibited values in the intervals of 80–97% and 81–96% in the training and test sets, respectively. On the other hand, when considering the drug sensitivity aspect (*ds*) of the different *P. falciparum* strains, *Sn*(*ds*) and *Sp*(*ds*) showed values between 90% and 94% in the training set, while *Sn*(*ds*) > 88% and *Sp*(*ds*) > 85% were reported for the test set. All the values of these local statistical metrics demonstrate that the PTML-MLP can accurately predict antiplasmodial activity across multiple *P. falciparum* strains and drug sensitivity labels (Supplementary Information S2).

In terms of the AD of the PTML-MLP, we previously mentioned (see Section 2.2) that a modification of the descriptors’ space approach was applied. In this sense, for each molecule present in the dataset and a defined *D*[*GBI*]*ej* index present in the PTML-MLP (see Table 1), a categorical value known as the local score of applicability domain (LSAD_*D*[*GBI*]*ej*) was calculated by comparing the *D*[*GBI*]*ej* value of that molecule with the maximum and minimum *D*[*GBI*]*ej* values. If the *D*[*GBI*]*ej* value for the molecule was within the boundaries of the maximum and minimum *D*[*GBI*]*ej* values, then the LSAD_*D*[*GBI*]*ej* associated with that molecule and considering that particular *D*[*GBI*]*ej* index was equal to one. If this condition was not satisfied, then the value LSAD_*D*[*GBI*]*ej* = 0 was obtained. Notice that this procedure was applied to each of the 25 *D*[*GBI*]*ej* indices in the PTML-MLP, which means that for each molecule, 25 LSAD_*D*[*GBI*]*ej* values were calculated. Then, for each molecule, the total score (TSAD) was determined. TSAD = 25 indicated that a molecule was within the AD of the PTML-MLP, while TSAD < 25 showed that the molecule was outside the AD, thus constituting an unreliable prediction. In the end, out of the 9595 cases of molecules in our dataset used to create the PTML-MLP, 9583 cases were within the AD (Supplementary Information S2).

**Figure 2:**
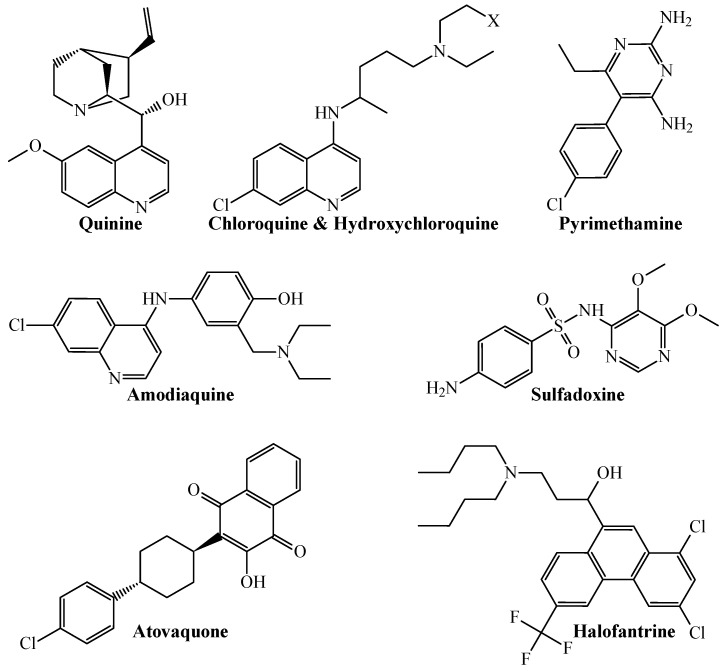
Chemical structures of different antimalarial drugs whose multi-strain antiplasmodial profile was correctly predicted by the PTML-MLP. Notice that X = H for chloroquine, while X = OH for hydroxychloroquine.

From a more chemical-oriented point of view, our PTML-MLP also demonstrated that it can correctly predict and classify the multi-strain antiplasmodial activity of well-established antimalarial drugs (Figure 2).

**Figure 3:**
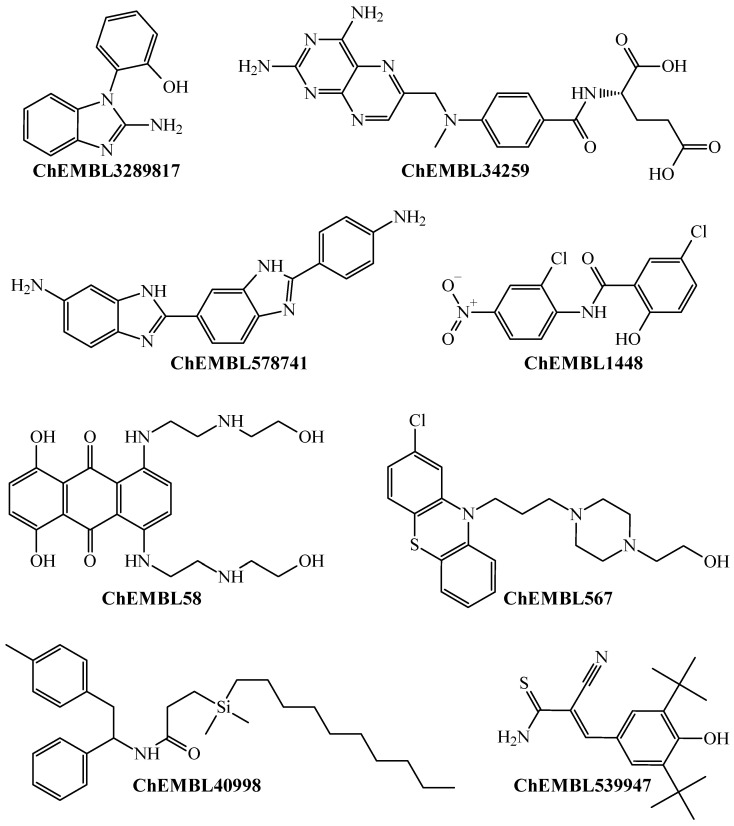
Chemicals containing different molecular patterns, which were predicted by the PTML-MLP to be versatile inhibitors against different *P. falciparum* strains.

The antimalarial drugs with multi-strain antiplasmodial activity correctly predicted by the PTML-MLP include (but are not limited to) those labeled in our dataset as ChEMBL170 (quinine), ChEMBL76 (chloroquine), ChEMBL1535 (hydroxychloroquine), ChEMBL36 (pyrimethamine), ChEMBL682 (amodiaquine), ChEMBL1539 (sulfadoxine), ChEMBL1450 (atovaquone), and ChEMBL1107 (halofantrine). Furthermore, our PTML-MLP was quite useful in identifying and predicting new molecular patterns (Figure 3), which are different from those present in the aforementioned antimalarial drugs.

Notice that Figure 3 presents a non-exhaustive list of molecules with different chemical structures. Because such molecules appear in the dataset, this means that they were experimentally assayed as multi-strain antiplasmodial agents and were also predicted by our PTML-MLP. We would like to highlight that some of these molecules are drugs approved by the Food and Drug Administration (FDA) with different therapeutic applications other than antimalarial activity. This is the case for the molecules labeled ChEMBL34259 and ChEMBL58, which are the FDA-approved drugs methotrexate and mitoxantrone, respectively. These are antineoplastic agents used in chemotherapies against different types of cancer. Furthermore, CHEMBL539947 (known as tyrphostin Ag-879) is a molecule with known anticancer activity [65,66]. Also, other FDA-approved drugs include ChEMBL1448 (niclosamide, an anthelmintic) and ChEMBL567 (perphenazine, an antipsychotic drug). All of this demonstrates that our PTML-MLP, in addition to having the capability to correctly identify and predict diverse molecular patterns with multi-strain antiplasmodial activity, can also be used as a computational tool in virtual screening scenarios to repurpose FDA-approved drugs.

### 3.2. The FBTD Approach: Interpretation of the PTML-MLP

**Figure 4:**
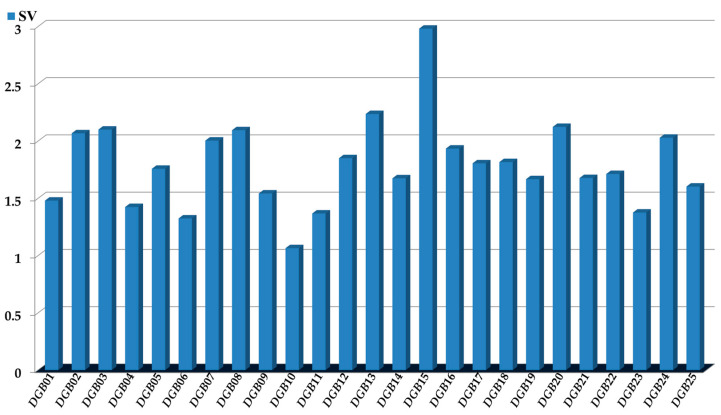
Sensitivity values (*SVs*): measures of the influence or discriminatory power of each of the *D*[*GBI*]*ej* indices present in the PTML-MLP model.

The physicochemical and structural interpretation of the *D*[*GBI*]*ej* indices present in the PTML-MLP is crucial to designing new molecules with the desired versatile inhibitory profile [30,46,56]. We considered four aspects; the first was the assessment of the significance of the *D*[*GBI*]*ej* indices (Figure 4) through the sensitivity values (*SVs*).

This means that the *D*[*GBI*]*ej* indices with the highest *SVs* were the ones for which, in addition to having a greater discriminatory power [67], the physicochemical properties and structural features characterized by them were most important in both the molecules of the dataset used to the build the PTML-MLP and any new molecule to be designed.

**Figure 5:**
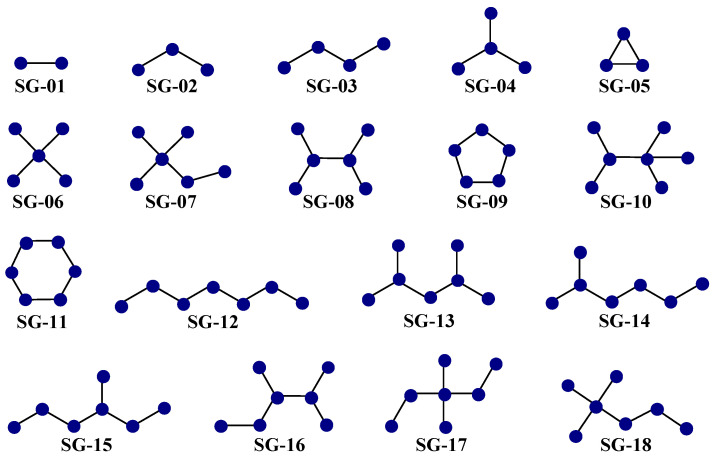
Most common subgraphs (SGs) characterized by the *D*[*GBI*]*ej* indices of the PTML-MLP. These generic fragments represent a wide variety of substructural moieties in the molecules.

The second aspect is that each of the 25 *D*[*GBI*]*ej* indices present in the PTML-MLP characterized the presence of different generic fragments (Figure 5), which are known as subgraphs (*SGs*).

Notice that each *SG* represents a generic fragment related to substructural moieties such as polar functional groups, aliphatic portions, and aliphatic and aromatic rings of different sizes.

The third aspect is the physicochemical information encoded by the *D*[*GBI*]*ej* indices; they maintain the same physicochemical and structural content as the topological indices from which they were calculated (see Equation (2)). Thus, the *D*[*GBI*]*ej* indices derived from *SM*(*PP*)*o* (see Section 2) describe how a defined physicochemical property is concentrated in different regions of a molecule while also being expressed as the number of times diverse *SGs* are present in that molecule [68,69,70,71,72,73]. The *D*[*GBI*]*ej* indices obtained from *Xv*(*SG*)*m* measure the molecular accessibility [74,75,76,77,78,79,80], i.e., the ability of the diverse *SGs* or fragments to effectively participate in both polar and non-polar interactions with other surrounding molecules (e.g., solvent molecules or components of a cellular membrane). The *D*[*GBI*]*ej* indices calculated from *e*(*SG*)*m* are quantitative contributions of the molecular volume of the different *SGs* and fragments [81,82,83,84,85].

Lastly, Table 3 illustrates the fourth aspect, i.e., how the value of each *D*[*GBI*]*ej* index should vary in the PTML-MLP to enhance the biological activity profile under study (in this study, the multi-strain antiplasmodial activity).

For each *D*[*GBI*]*ej* index, two average values were computed: one for the cases annotated and correctly classified as active and the other for the cases labeled and correctly classified as inactive [30,46,56]. When comparing the two averages, if the value considered active is higher, then this means that the value of that particular *D*[*GBI*]*ej* index should be increased to enhance the multi-strain antiplasmodial activity; otherwise, the value of that *D*[*GBI*]*ej* index will have to be decreased. We would like to emphasize that only correctly annotated and classified cases were used to compute the aforementioned *D*[*GBI*]*ej*-based averages. This ensured a clean and reliable interpretation of the trends in the *D*[*GBI*]*ej* indices, tightly aligned with the actual statistical quality and predictive performance of the PTML-MLP.

There were 12 *D*[*GBI*]*ej* indices derived from *SM*(*PP*)*o* in our PTML-MLP. In this sense, *DGB*01 and *DGB*03 characterized the increase in polarizability. Notice that *DGB*01 focuses on the SG-04 and SG-06 subgraphs; SG-04 represented the presence of sulfur or a halogen (with Cl, Br, and I being preferred over F) attached to a ring, as well as two rings connected by a single bond or forming a condensed aromatic system. For SG-06, this involved CZ_3_ and OCZ_3_ moieties, where Z can be a methyl or a halogen (with Cl, Br, and I preferred over F). Notice that SG-06 contains several SG-04 subgraphs. In contrast, *DGB*03 is a measure of the global polarizability of a molecule (SG-01), favoring the presence of aromatic rings and heteroaromatic rings, as well as sulfur, nitrogen from secondary and tertiary aromatic amines, halogens other than fluorine, and heteroaliphatic rings. We would like to highlight that in terms of importance in the PTML-MLP, *DGB*01 and *DGB*03 ranked 20th and 4th, respectively.

On the other hand, *DGB*02 and *DGB*17 indicate the augmentation of the hydrophobicity. Notice that *DGB*02 describes this property in the SG-04 subgraphs (e.g., the CZ_3_ and OCZ_3_ moieties mentioned above, tertiary amines, two rings connected by a single bond or forming a condensed aromatic system, and any sulfur and halogen atom attached to rings or as part of an aliphatic ramification) as well as the SG-05 subgraphs (three-membered rings, particularly oxirane and thiirane). In the case of *DGB*17, this measures the global hydrophobicity (SG-01 subgraph, with each bond of a molecule being hydrophobically favored by the presence of sulfur and halogen atoms, as well as aromatic carbons (except those containing nitrogen or bound to a nitrogen or oxygen atom)). *DGB*02 and *DGB*17 ranked 6th and 12th, respectively, among the *D*[*GBI*]*ej* indices in the PTML-MLP.

In the case of *DGB*04, this indicates the increase in the value of the charge through each bond of the molecule (SG-01 subgraph), meaning that functional groups having a high electronic density (e.g., hydroxyl or amino) should be reduced, thus favoring the presence of halogens such as Cl, Br, and I. Simultaneously, *DGB*04 (ranking as 21st most important among the *D*[*GBI*]*ej* indices) favors heteroaromatic rings with pyridinic nitrogen atoms (particularly pyridine, pyridazine, and pyrimidine). Heteroaliphatic rings lacking nitrogen and oxygen (e.g., tetrahydro-2H-thiopyran) are also quite suitable.

The *D*[*GBI*]*ej* indices *DGB*09 and *DGB*18 characterized the diminution of the polar surface area. In the case of *DGB*09 (the 19th most influential *D*[*GBI*]*ej* index), this focuses on the SG-04 and SG-06 subgraphs. For SG-04, the number of carbonyl, amide, urea, and sulfoxide groups should be reduced as much as possible; alkoxy, phenoxy, alkylamino, pyridine-3-olate, and arylamino are more suitable due to their lower polar surface areas. The same line of thinking applies to polar groups represented by the SG-06 subgraph; the previously described functional groups CZ_3_ and OCZ_3_ (Z can be only halogen or methyl) should be present only once when possible. The *D*[*GBI*]*ej* index *DGB*18 (ranking 11th) focused on the diminution of the global polar surface area of each bond (SG-01 subgraph) of a molecule. Thus, the number of functional groups with high polar surface areas (e.g., amide, sulfone, sulfonamide, and sulfoxide) should be decreased.

The decrease in the atomic weight is described in the PTML-MLP by *DGB*10 and *DGB*19. It should be highlighted that *DGB*10 (ranking 25th among the *D*[*GBI*]*ej* indices) characterized the presence of many fragments in which the atomic weight of the atoms should be diminished. This was the case for the subgraphs of SG-04 (presence of two rings connected by a single bond or forming a condensed aromatic system ring systems and containing mainly carbon, nitrogen, and oxygen, as well as fluorine and, to a lesser degree, chlorine), SG-05 (three-membered rings, mainly cyclopropane), SG-06 (favoring the CZ_3_ and OCZ_3_ groups, with Z being preferably fluorine or methyl, respectively), SG-07 (e.g., trifluoromethoxy or tert-butoxy groups attached to any atom), SG-08 (focused on two rings connected by a single bond or forming a condensed aromatic system containing only carbon and nitrogen), and SG-09 (five-membered rings containing only carbon, nitrogen, and oxygen). Regarding *DGB*19 (being the 17th most important *D*[*GBI*]*ej* index), this is a measure of the molecular weight. Therefore, to favorably decrease its value, the number of atoms different from carbon, nitrogen, oxygen, and fluorine should be greatly reduced.

The *D*[*GBI*]*ej* index *DGB*14, which ranked 16th in the PTML-MLP model, described the increase in the bond distance in each bond of the molecule, where the presence of atoms such as halogens (other than fluorine) and sulfur is favorable. At the same time, the *D*[*GBI*]*ej* indices *DGB*15 and *DGB*16 described the diminution of the dipole moment. In the case of *DGB*15, which is the most important *D*[*GBI*]*ej* index in the PTML-MLP, this described the same subgraph (SG-01) as explained for *DGB*18 above. However, while *DGB*18 gave priority to the decrease in polar groups to diminish the potential interactions via hydrogen bonds, *DGB*15 focused more on decreasing the number of polar groups to favor non-polar interactions (London dispersion forces through carbons and halogens). Thus, the presence of any moiety containing a carbonyl, amide, thiocarbonyl, nitrile, sulfoxide, sulfone, sulfonamide, thioether, or nitro group should be avoided. At the same time, *DGB*16 (ranking ninth) characterized the same subgraphs and fragments as *DGB*10. For this reason, to favorably diminish the dipole moment, the *D*[*GBI*]*ej* index *DGB*16 prioritized the increment of the number of rings (both aliphatic or aromatic) connected through a single bond or forming a condensed system (SG-04). For this same subgraph, nitrogen (both as amino group and as part of a ring), as well as the hydroxyl, alkoxy, phenoxy, and pyridine-3-olate groups, are also suitable. Through the *D*[*GBI*]*ej* index *DGB*16, the dipole moment could also be favorably diminished in other subgraphs such as SG-05 (three-membered rings, e.g., cyclopropane), SG-06 (priority is given to tert-butyl and tert-butoxy), SG-07 (tert-butoxy is preferred), SG-08 (condensed heteroaromatic system), and SG-09 (five-membered rings with low polarity, e.g., cyclopentane and pyrrole).

The *D*[*GBI*]*ej* indices *DGB*11, *DGB*12, *DGB*20, *DGB*21, and *DGB*22 measured the influence of molecular accessibility based on the occurrence of polar and non-polar interactions in different regions of a molecule, and they ranked 23rd, 10th, 3rd, 15th, and 14th, respectively. In this sense, *DGB*11 indicated the decrease in the number of five-membered rings. If present, then an aromatic five-membered ring (e.g., pyrrole or imidazole) would be preferred over its aliphatic counterparts, and it should have substitutions in more than two positions. Simultaneously, *DGB*12 characterized the diminution of the number of many large fragments or subgraphs (SG-13, SG-14, SG-15, SG-16, SG-17, and SG-18). Because these fragments and subgraphs are quite common, they should be based on polar functional groups and moieties that are formed only by carbon, nitrogen, and oxygen. In all of these fragments, aliphatic chains are preferred the least, while structural moieties containing aromatic rings (whether fused or connected through a single bond) are favored. The diminution of linear fragments formed by three bonds (SG-03 subgraph) was described by *DGB*20, and therefore, when present, such fragments should contain only carbon, nitrogen, and oxygen. In the case of *DGB*21, this characterized the diminution of fragments or functional groups based on the SG-04 subgraph. Notice that the SG-04 subgraph representing ramifications (and substitution in rings) should also be reduced. If present, SG-04 subgraphs should appear in the form of hydroxyl, alkoxy, phenoxy, amino, alkylamino, and arylamino groups attached to rings (or secondary carbons). When considering *DGB*22, it should be pointed out that in the case of the SG-11 subgraphs, the presence of six-membered rings is highly desirable, where six-membered aliphatic rings (including those containing heteroatoms) are preferred over their aromatic counterparts.

The *D*[*GBI*]*ej* indices *DGB*05, *DGB*06, *DGB*07, *DGB*08, *DGB*13, *DGB*23, and *DGB*24, which were graded 13th, 24th, 8th, 5th, 2nd, 22nd, and 7th among the most influential indices in the PTML-MLP, respectively, represent steric aspects, constituting measures of the volume in different regions of a molecule. In the case of *DGB*05, this described the decrease in the global molecular volume (SG-01 subgraph, each bond of the molecule), thus supporting the presence of ramifications and polysubstituted rings. In contrast, *DGB*06 favored an increment in the volume by increasing the number of linear fragments in a molecule (SG-02 subgraph), while *DGB*07 exerted a similar effect to *DGB*06 but focused on linear fragments based on the SG-12 subgraphs. Notice that *DGB*06 and *DGB*07 did not favor the presence of ramifications. Therefore, if ramifications are present, then they are preferred in the periphery of a molecule. Furthermore, *DGB*08 indicated the increase in the number of six-membered rings, with emphasis on those where the number of substituents was low (not more than two). In the case of *DGB*13, this involved the increase in the number of ramifications based on the SG-06 subgraph. This means that the presence of the aforementioned functional groups CZ_3_ and OCZ_3_ (with Z being mainly methyl or halogen) is favorable. For the case of *DGB*23, this indicated the decrease in the number of moieties containing the SG-10 subgraph. Notice that SG-10 is composed of SG-02 and SG-06, which means that if SG-06 is present, then the functional groups of the type OCZ_3_ (with Z being methyl or halogen) are highly suitable. The *D*[*GBI*]*ej* index *DGB*24 characterized the diminution of the number of the same types of fragments as discussed above for *DGB*12. However, *DGB*24 described the influence of the volume of those fragments (instead of the molecular accessibility characterized by *DGB*12). Finally, the *D*[*GBI*]*ej* index *DGB*25, which ranked 18th in the PTML-MLP, described the shape of a molecule by decreasing the number of SG-03 subgraphs, which should be part of rings, particularly two or three rings interconnected by single bonds or forming a condensed or fused aromatic system.

### 3.3. Combining the PTML-MLP and FBTD to Enable the Design of Multi-Strain Antiplasmodial Inhibitors

Only the joint interpretation of the *D*[*GBI*]*ej* indices in the PTML-MLP model allowed us to design new molecules. In this sense, we would like to point out that, in its essence, the joint interpretation of all the *D*[*GBI*]*ej* indices is to design molecules that maximize the effect of all the physicochemical properties and structural features in the PTML-MLP. This means that the design molecule will contain a series of fragments and moieties whose presences favorably and simultaneously vary (either increasing or decreasing) the values of several *D*[*GBI*]*ej* indices in the PTML-MLP. Thus, the joint interpretations used to design the molecules indicated that several six-membered rings (probably three or four) should be present in the chemical structure of a molecule, either forming a fused ring system or one connected through a single- or a two-bond fragment containing a heteroatom (nitrogen or oxygen). Also, these six-membered rings should be polysubstituted.

**Figure 6:**
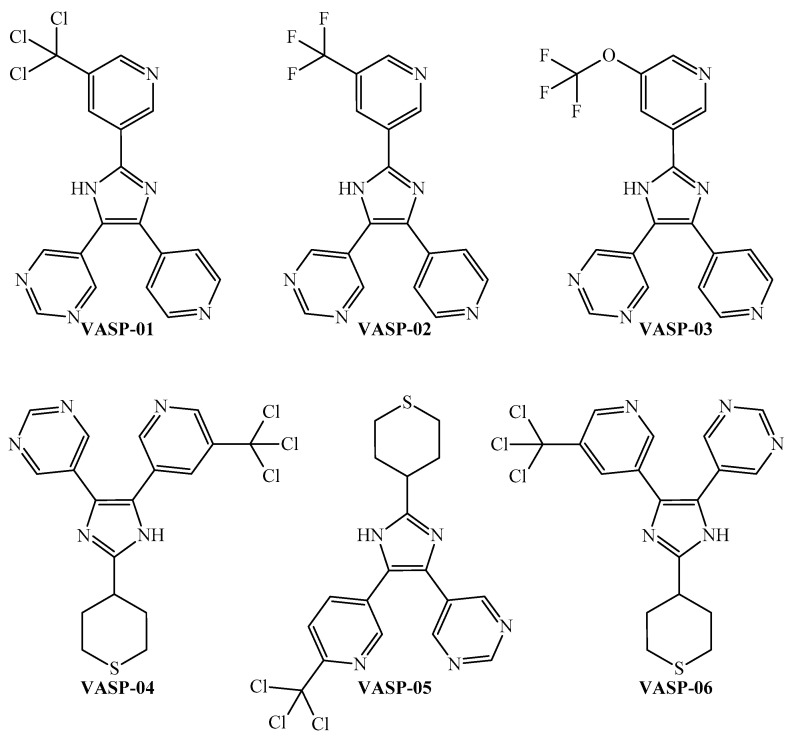
New molecules designed through the application of the FBTD approach to the PTML-MLP.

The peripheral parts of the molecule are incredibly important. Notice that although most of these six-membered rings in a molecule should be heteroaromatic (pyridine, pyridazine, and pyrimidine), a polysubstituted aromatic ring may also be suitable. In the case of an aliphatic ring (preferably a sulfur-containing cycle), when present, this should be in the periphery of the molecule. The peripheral part of the molecule is also the region where halogens (the sum should not be more than four) or sulfur (not more than one) should be placed. For ramifications, functional groups such as CZ_3_ and OCZ_3_ (where Z is methyl, fluorine, or chlorine) are expected to also appear in the peripheral part of the molecule. On the other hand, the presence of a five-membered ring is desirable when this ring is heteroaromatic (pyrrole, imidazole, oxazole, etc.). By rigorously following the joint interpretation as a guideline, we designed six molecules (Figure 6) and predicted their multi-strain antiplasmodial activity using the PTML-MLP.

Notice that if a molecule is predicted by the PTML-MLP to be active with a probability (ProbAct) value higher than 50% against a given *P. falciparum* strain, then this molecule can be considered to exhibit antiplasmodial activity against that particular strain. Almost all of the predictions for the six designed molecules fell within the AD of the PTML-MLP (Supplementary Information S3). The only exception was the prediction of the molecule VASP-03 against the *P. falciparum* (FCB), which yielded TSAD = 24 (instead of the ideal value of TSAD = 25). In any case, the results from Table 4 indicate that all of the designed molecules satisfied this condition against several *P. falciparum* strains. This means that the six designed molecules were predicted by the PTML-MLP to be multi-strain antiplasmodial inhibitors, thus virtually displaying IC_50_ ≤ 500 nM (the activity cutoff used to develop the PTML-MLP) against three or more *P. falciparum* strains. It is also important to point out that despite the relatively subtle structural differences among the designed molecules, there was great variation in the ProbAct values across different *P. falciparum* strains. This confirms the great discriminatory power and meaningful chemistry-driven information of the *D*[*GBI*]*ej* indices in the PTML-MLP, favorably enabling them to capture relevant structural differences not only among heterogenous groups of chemicals (as demonstrated by the statistical performance of the PTML-MLP in Section 3.1, including Figure 2 and Figure 3) but also within a series of structurally related chemicals (as in the case of the six designed molecules).

A simple inspection of Table 4 and Figure 6 indicates that the six designed molecules are divided into two subfamilies of 2,4,5-trisubstituted imidazoles. In the first subfamily, composed of VASP-01, VASP-02, and VASP-03, all of the rings are heteroaromatic, and the main chemical variation is the substitution of the 5-(substituted)pyridin-3-yl moiety, where the order of suitability to enhance the multi-strain antiplasmodial activity is trichloromethyl > trifluoromethoxy > trifluoromethyl. In this sense, the key role of the trichloromethyl fragment in the discovery and design of antiplasmodial molecules has been strongly supported by experimental evidence [86,87]. Likewise, biological assays for determination of the antiplasmodial activity have permitted experimentally corroborating the relative superiority of trifluoromethoxy over trifluoromethyl [88].

Therefore, for the design of the second subfamily of 2,4,5-trisubstituted imidazoles (formed by VASP-04, VASP-05, and VASP-06), we maintained the trichloromethyl group while introducing the tetrahydro-2H-thiopyran moiety in a peripheral part of the molecules. By performing these two chemical modifications, the designed molecules of the second subfamily exhibit higher ProbAct values than those from the first subfamily, with the former being predicted to display multi-strain antiplasmodial activity against the nine *P. falciparum* strains reported in this work.

Notice that we are not considering all of the possible chemical variations or modifications of the chemical structures within the two subfamilies of 2,4,5-trisubstituted imidazoles; we are focusing only on specific modifications through the use of certain functional groups to demonstrate the ability of the PTML model (through the *D*[*GBI*]*ej* indices) to efficiently discriminate between even small chemical modifications. In any case, the six designed molecules exhibited great potential to inhibit different *P. falciparum* strains with different degrees of resistance to current antimalarial drugs.

We wanted to assess whether the six designed molecules had some novelty from a chemistry-based point of view. For this, the chemical structures of the designed molecules were searched for in several well-known large databases such as ChEMBL [47,48,89], ZINC [90], and eMolecules [91]. These online repositories, in addition to containing chemical (and, in some cases, bioactivity profile) information on organic molecules, allow searching for chemical similarity by assessing the Tanimoto coefficient (*T*) [92]. Although the widely accepted cutoff of chemical similarity is *T* ≥ 0.85 [92,93], here we used a slightly more rigorous cutoff (*T* ≥ 0.8) to consider potential chemical similarity when comparing the structures of our six designed molecules with the structures of the molecules present in the aforementioned databases.

None of the chemical structures of the designed molecules were reported in these databases. This means that no exact matches were found. Furthermore, the similarity searches using *T* ≥ 0.8 failed to retrieve any molecules structurally related to our designed molecules (from VASP-01 to VASP-06) in these databases. This strongly supports the chemical novelty and scaffold uniqueness of these designed molecules. At the same time, given that the designed molecules fell into the 2,4,5-trisubstituted imidazole class but with non-reported, multi-substituted heteroaromatic moieties and trichloromethyl and tetrahydrothiopyran fragments, their novelty is not only structural but also pharmacologically contextual, as no such combinations have been reported in the antiplasmodial or wider bioactivity domains.

All of this suggests that the combined use of our PTML-MLP FBTD approach can lead to new scaffolds and scaffold-based chemicals virtually displaying multi-strain antiplasmodial activity which can be considered for future organic synthesis and bioactivity evaluation (in this case, antiplasmodial activity).

### 3.4. Druglikeness of the Designed Molecules

Regarding the six designed molecules, in addition to assessing their chemistry-based novelty and their encouraging virtual multi-strain antiplasmodial profiles against different *P. falciparum* strains, we explored the druglikeness. For this, we used the software AlvaDesc v1.0.22 [94], and we computed a series of physicochemical properties (Table 5).

It is important to highlight that physicochemical properties such as the molecular weight (MW), total number of atoms (TNA), number of rotatable bonds (NRB), numbers of atoms behaving as hydrogen bond donors (HBDs) or acceptors (HBAs), molar refractivity (MR), polar surface area (PSA), and the logarithm of the n-octanol-water partition coefficient (MLOGP and ALOGP) are closely related to druglikeness-based standards such as the Lipinski rule of five [95], Ghose’s guidelines [96], and Veber’s filter [97]. In this sense, MW < 500 Da, HBD ≤ 5, and HBA ≤ 10, in addition to the MLOGP and ALOGP being below 5, should be property values that a molecule should have to comply with Lipinski’s rule of five. Furthermore, for the case of Ghose’s guidelines, a molecule should possess an MLOGP and ALOGP in the range from −0.4 to +5.6, with 40 ≤ MR ≤ 130, 180 ≤ MW ≤ 480, and 20 ≤ TNA ≤ 70. To satisfy Veber’s filter, a molecule should exhibit PSA ≤ 140 and NRB ≤ 10. The examination of all these physicochemical property values depicted in Table 5 shows that the six designed molecules complied with these three druglikeness-based standards, thus highlighting their adequate druglikeness.

To further expand our discussion in terms of druglikeness, we predicted many absorption, distribution, metabolism, elimination, and toxicity (ADMET) endpoints of the six designed molecules. For this, we employed the ADMETLab web server [98], which allowed us to estimate 31 ADMET endpoints (Supplementary Information S4). In summary, the six designed molecules demonstrated favorable pharmacokinetic and safety profiles. Despite being predicted to exhibit a relatively low solubility, the six designed molecules displayed acceptable predicted values for Caco-2 cell permeability, human intestinal absorption, and oral bioavailability. Regarding the distribution phase, most of them were predicted to have plasma protein binding (PPB) levels below 90%, and their volumes of distribution fell within the optimal range of 0.04–20 L/kg. The designed molecules were predicted to be able to penetrate the blood–brain barrier (BBB), a critical trait for therapeutic agents targeting cerebral malaria.

Regarding metabolism, the designed molecules showed varying degrees of interaction potential with the major cytochrome P450 (CYP) enzymes (particularly CYP1A2, CYP3A4, CYP2C9, CYP2C19, and CYP2D6). These compounds were mainly estimated as CYP inhibitors and non-CYP substrates. As for the elimination profiles, the predicted clearance rates for the six designed molecules were low, and the half-lives were relatively high.

When assessing toxicity, one concern was the potential for inhibition of the hERG channel, which may lead to cardiotoxic effects. However, we should highlight that the ADMET web server used a stringent threshold (IC_50_ < 40 µM), which is more conservative than the commonly used cutoff of IC_50_ ≤ 10 µM for defining significant hERG inhibition. At the same time, some caution should be taken since the designed molecules were predicted to exhibit hepatotoxicity. With regard to mutagenicity, skin sensitization, and acute in vivo toxicity, the compounds were generally considered safe.

Altogether, the predicted ADMET profiles suggest that the six designed molecules possess a sufficiently promising pharmacokinetic and safety profile, justifying future synthesis and experimental investigation in the context of early-stage antimalarial discovery.

## 4. Conclusions

Despite the current intensive search for novel antiplasmodial agents, new chemicals capable of fighting against *P. falciparum* malaria are presently needed. Consequently, in silico methodologies should focus more efforts on the early discovery of molecules exhibiting great versatility when inhibiting multiple *P. falciparum* strains, thus contributing to avoiding drug resistance issues, including MDR strains. The findings presented in this study indicate that the integrated use of a PTML-MLP and the FBTD approach can enable a deeper interpretation of the diverse physicochemical properties and structural aspects associated with the appearance and enhancement of multi-strain antiplasmodial activity. This led to the computational design of new molecules seemingly exhibiting both chemical novelty and multi-strain antiplasmodial profile. The unified application of PTML modeling and FBTD opens new horizons for the chemistry-driven in silico generation of new scaffolds and scaffold-based compounds with multi-strain antiplasmodial activity, which could be expanded to other microbial diseases beyond malaria.

## Figures and Tables

**Table 1:** The *D*[*GBI*]*ej* indices: symbols and concepts.

Codes ^a,b,c^	Symbols	Concepts
*DGB*01	*D*[*SM*(*Mol*)4]*tg*	Multi-label graph index derived from the bond-based spectral moment of the 4th order, weighted by atom-based molar refractivities.
*DGB*02	*D*[*NSM*(*Hyd*)3]*tg*	Multi-label graph index derived from the normalized bond-based spectral moment of the 3rd order, weighted by atom-based hydrophobicities.
*DGB*03	*D*[*NSM*(*Mol*)1]*tg*	Multi-label graph index derived from the normalized bond-based spectral moment of the 1st order, weighted by atom-based molar refractivities.
*DGB*04	*D*[*NSM(Gas*)1]*tg*	Multi-label graph index derived from the normalized bond-based spectral moment of the 1st order, weighted by Gasteiger-Marsili atomic charges.
*DGB*05	*D*[*Ne*(*P*)1]*tg*	Multi-label graph index derived from the normalized bond-based connectivity of the 1st order, containing only path subgraphs.
*DGB*06	*D*[*Ne*(*P*)2]*tg*	Multi-label graph index derived from the normalized bond-based connectivity of the 2nd order, containing only path subgraphs.
*DGB*07	*D*[*Ne*(*P*)6]*tg*	Multi-label graph index derived from the normalized bond-based connectivity of the 6th order, containing only path subgraphs.
*DGB*08	*D*[*Ne*(*Ch*)6]*tg*	Multi-label graph index derived from the normalized bond-based connectivity of the 6th order, containing only cycle (ring) subgraphs.
*DGB*09	*D*[*SM*(*Psa*)4]*ds*	Multi-label graph index derived from the bond-based spectral moment of the 4th order, weighted by atom-based polar surface areas.
*DGB*10	*D*[*SM*(*Ato*)7]*ds*	Multi-label graph index derived from the bond-based spectral moment of the 7th order, weighted by atomic weights.
*DGB*11	*D*[*Xv*(*Ch*)5]*ds*	Multi-label graph index derived from the atom-based valence connectivity of the 5th order, containing only cycle (ring) subgraphs.
*DGB*12	*D*[*Xv*(*PC*)6]*ds*	Multi-label graph index derived from the atom-based valence connectivity of the 6th order, containing only path-cluster subgraphs.
*DGB*13	*D*[*e*(*C*)4]*ds*	Multi-label graph index derived from the bond-based connectivity of the 4th order, containing only cluster subgraphs.
*DGB*14	*D*[*NSM*(*Std*)1]*ds*	Multi-label graph index derived from the normalized bond-based spectral moment of the 1st order, weighted by the standard bond distances.
*DGB*15	*D*[*NSM*(*Dip*)1]*ds*	Multi-label graph index derived from the normalized bond-based spectral moment of the 1st order, weighted by the bond dipole moments.
*DGB*16	*D*[*NSM*(*Dip*)7]*ds*	Multi-label graph index derived from the normalized bond-based spectral moment of the 7th order, weighted by the bond dipole moments.
*DGB*17	*D*[*NSM*(*Hyd*)1]*ds*	Multi-label graph index derived from the normalized bond-based spectral moment of the 1st order, weighted by atom-based hydrophobicities.
*DGB*18	*D*[*NSM*(*Psa*)1]*ds*	Multi-label graph index derived from the normalized bond-based spectral moment of the 1st order, weighted by atom-based polar surface areas.
*DGB*19	*D*[*NSM*(*Ato*)1]*ds*	Multi-label graph index derived from the normalized bond-based spectral moment of the 1st order, weighted by atomic weights.
*DGB*20	*D*[*NXv*(*P*)3]*ds*	Multi-label graph index derived from the normalized atom-based valence connectivity of the 3rd order, containing only path subgraphs.
*DGB*21	*D*[*NXv*(*C*)3]*ds*	Multi-label graph index derived from the normalized atom-based valence connectivity of the 3rd order, containing only cluster subgraphs.
*DGB*22	*D*[*NXv*(*Ch*)6]*ds*	Multi-label graph index derived from the normalized atom-based valence connectivity of the 6th order, containing only cycle (ring) subgraphs.
*DGB*23	*D*[*Ne*(*C*)6]*ds*	Multi-label graph index derived from the normalized bond-based connectivity of the 6th order, containing only cluster subgraphs.
*DGB*24	*D*[*Ne*(*PC*)6]*ds*	Multi-label graph index derived from the normalized bond-based connectivity of the 6th order, containing only path-cluster subgraphs.
*DGB*25	*D*[*NK*(*Alpha*)3]*ds*	Multi-label graph index derived from the normalized alpha-modified shape descriptor of the 3rd order, containing only path subgraphs.

^a^ The codes for the *D*[*GBI*]*ej* indices will be used throughout the entire manuscript. ^b^ For the *D*[*GBI*]*ej* indices containing the symbol “*SM*”, the order (mentioned above with the notation “*o*”) is the maximum number of bonds that a fragment can have without considering bond multiplicity. For the *D*[*GBI*]*ej* indices containing the symbols “*Xv*” and “*e*”, the order (mentioned above with the notation “*m*”) is the exact number of bonds (without considering multiplicity) present in a fragment. ^c^ The notation *tg* indicates that the *D*[*GBI*]*ej* indices depend on the chemical structure and the specific *P. falciparum* strain. Likewise, *ds* indicates that the *D*[*GBI*]*ej* indices depend on the chemical structure and whether a *P. falciparum* strain is sensitive or resistant to current antimalarial drugs.

**Table 2:** Global metrics of performance associated with the PTML-MLP.

SYMBOLS ^a^	Training Set	Test Set
*N*_Active_	3613	1204
*TP*	3393	1074
*Sn*	93.91%	89.20%
*N*_Inactive_	3584	1194
*TN*	3260	1029
*Sp*	90.96%	86.18%
*nMCC*	0.925	0.877

^a^*N*_Active_ = number of cases annotated as active; *N*_Inactive_ = number of cases annotated as inactive; *TP* = true positive; *TN* = true negative; *Sn* = sensitivity (percentage of cases correctly predicted as active); *Sp* = specificity (percentage of cases correctly predicted as inactive); *nMCC* = normalized Matthews correlation coefficient.

**Table 3:** Variation in the values of the different *D*[*GBI*]*ej* indices.

Codes ^a^	Average Values		Tendency ^b^
	Active	Inactive	
*DGB*01	8.220 × 10^−3^	−1.054 × 10^−1^	Increase
*DGB*02	1.195 × 10^−2^	−5.387 × 10^−2^	Increase
*DGB*03	−2.959 × 10^−4^	−1.455 × 10^−2^	Increase
*DGB*04	5.718 × 10^−3^	−6.431 × 10^−2^	Increase
*DGB*05	−9.300 × 10^−3^	1.488 × 10^−1^	Decrease
*DGB*06	3.953 × 10^−3^	−2.272 × 10^−1^	Increase
*DGB*07	6.967 × 10^−3^	−2.938 × 10^−1^	Increase
*DGB*08	−7.765 × 10^−3^	−8.953 × 10^−2^	Increase
*DGB*09	3.773 × 10^−3^	1.589 × 10^−1^	Decrease
*DGB*10	1.687 × 10^−4^	1.049 × 10^−1^	Decrease
*DGB*11	−4.437 × 10^−4^	2.400 × 10^−2^	Decrease
*DGB*12	4.613 × 10^−3^	1.039 × 10^−2^	Decrease
*DGB*13	1.253 × 10^−2^	−4.819 × 10^−2^	Increase
*DGB*14	2.450 × 10^−4^	−1.427 × 10^−1^	Increase
*DGB*15	−1.374 × 10^−3^	2.750 × 10^−1^	Decrease
*DGB*16	2.155 × 10^−3^	1.896 × 10^−1^	Decrease
*DGB*17	4.187 × 10^−3^	−1.435 × 10^−1^	Increase
*DGB*18	5.651 × 10^−5^	2.241 × 10^−1^	Decrease
*DGB*19	8.620 × 10^−3^	1.171 × 10^−1^	Decrease
*DGB*20	2.873 × 10^−3^	4.588 × 10^−2^	Decrease
*DGB*21	4.693 × 10^−3^	1.153 × 10^−1^	Decrease
*DGB*22	4.674 × 10^−3^	−1.728 × 10^−1^	Increase
*DGB*23	2.511 × 10^−3^	1.447 × 10^−1^	Decrease
*DGB*24	1.987 × 10^−3^	2.057 × 10^−3^	Decrease
*DGB*25	1.196 × 10^−3^	8.020 × 10^−2^	Decrease

^a^ The codes are the same as those present in Table 1. ^b^ This refers to the variation (decrease or increase in the value of a defined *D*[*GBI*]*ej* index).

**Table 4:** Predictions of multi-strain antiplasmodial activity for the six designed molecules.

*tg* ^a^	*ds* ^b^	ProbAct (%) ^c,d^					
		VASP-01	VASP-02	VASP-03	VASP-04	VASP-05	VASP-06
*P. falciparum* (7G8)	Drug-resistant	56.83	44.11	46.14	74.24	73.70	74.24
*P. falciparum* (Dd2)	Drug-resistant	56.91	45.83	49.81	82.26	81.00	82.26
*P. falciparum* (D6)	Drug-sensitive	64.45	59.43	65.36	83.91	82.58	83.91
*P. falciparum* (3D7)	Drug-sensitive	64.38	49.00	57.75	82.21	81.12	82.21
*P. falciparum* (W2)	Drug-resistant	66.42	38.16	45.59	80.33	79.08	80.33
*P. falciparum* (D10)	Drug-sensitive	69.34	44.91	51.04	74.30	72.86	74.30
*P. falciparum* (FCB)	Drug-resistant	61.36	87.06	87.14	64.44	66.67	64.44
*P. falciparum* (K1)	Drug-resistant	69.60	41.70	47.34	78.69	77.99	78.69
*P. falciparum* (NF54)	Drug-sensitive	66.98	51.01	55.71	79.98	79.18	79.98

^a^ The element tg refers to a specific *P. falciparum* strain. ^b^ The element ds indicates whether a defined *P. falciparum* strain is sensitive or resistant to current antimalarial drugs. ^c^ The symbol “VASP-” is the code associated with each designed molecule. ^d^ The term “ProbAct” represents the probability value (expressed as a percentage) predicted by the PTML-MLP to classify a molecule as active.

**Table 5:** Druglikeness-related physicochemical properties calculated for the designed molecules.

ID	Physicochemical Properties ^a^								
	MW	TNA	NRB	HBD	HBA	MR	PSA	MLOGP	ALOGP
VASP-01	417.70	38	3	1	5	106.26	80.24	2.9041	2.8515
VASP-02	368.35	38	3	1	8	91.760	80.24	2.568	2.2614
VASP-03	384.35	39	4	1	9	93.361	89.47	1.8088	3.439
VASP-04	440.81	43	3	1	4	113.17	92.65	3.6301	3.4394
VASP-05	440.81	43	3	1	4	112.80	92.65	3.6301	3.8679
VASP-06	440.81	43	3	1	4	113.17	92.65	3.6301	3.4394

**^a^** The symbols used for the different physicochemical properties are as follows: MW = molecular weight (expressed in daltons); TNA = total number of atoms in a molecule (including hydrogen atoms); NRB = number of rotatable bonds; HBD = number of atoms participating in hydrogen bonds as donors; HBA = number of atoms participating in hydrogen bonds as acceptors; MR = molar refractivity (expressed in cm^3^/mol), estimated according to the Ghose–Crippen atomic and fragment contributions; PSA = topological polar surface area (expressed in Å^2^), estimated from atomic and fragment contributions for functional groups containing nitrogen, oxygen, fluor, sulfur, and phosphorus; MLOGP = logarithm of the n-octanol-water partition coefficient, estimated according to Moriguchi’s method; ALOGP = logarithm of the n-octanol-water partition coefficient, estimated according to the Ghose–Crippen atomic and fragment contributions.

## Data Availability

The original contributions presented in this study are included in the article/Supplementary Material. Further inquiries can be directed to the corresponding author.

## Supplementary Materials

The following supporting information can be downloaded at. https://www.mdpi.com/article/10.3390/microorganisms13071620/s1. Supplementary Information S1: Graph-based indices (*GBIs*) (including different families of topological indices (*TIs*) and their normalization-like counterparts (*NTIs*)), averages *avg*[*GBI*]*ej*, and standard deviation values *sdv*[*GBI*]. Supplementary Information S2: *D*[*GBI*]*ej* indices, classification results, local metrics, and applicability domain. Supplementary Information S3: Graph-based indices (*GBIs*) (including different families of topological indices (*TIs*) and their normalization-like counterparts (*NTIs*)), *D*[*GBI*]*ej* indices, classification results, and applicability domain for the designed molecules. Supplementary Information S4: Predicted ADMET endpoints of the designed molecules.
