# Supplementary material for: In Silico Approach for Early Antimalarial Drug Discovery: De Novo Design of Virtual Multi-Strain Antiplasmodial Inhibitors
Source: Microorganisms. 2025 Jul 9;13(7):1620. doi: 10.3390/microorganisms13071620 (PMC12300622; doi:10.3390/microorganisms13071620)
Supplement: Supplementary file 1 [file microorganisms-13-01620-s001.zip › Supplementary Information S4 (Antiplasmodial_P. falciparum)_OK.pdf]

In Silico Approach for Early Antimalarial Drug Discovery: *De Novo* Design of Virtual Multi-Strain Antiplasmodial Inhibitors

Valeria V. Kleandrova, M. Natália D. S. Cordeiro and Alejandro Speck-Planche *

LAQV@REQUIMTE/Department of Chemistry and Biochemistry, Faculty of Sciences, University of Porto, 4169-007, Porto, Portugal

***Corresponding Author:** Alejandro Speck-Planche (alejspivanovich@gmail.com);
ORCID ID: Valeria V. Kleandrova (https://orcid.org/0000-0002-1928-853X).
M. Natália D. S. Cordeiro (https://orcid.org/0000-0003-3375-8670).
Alejandro Speck-Planche (https://orcid.org/0000-0002-9544-9016).

The present supplementary material file contains the different ADMET endpoints predicted for the six designed molecules. These endpoints were estimated by the ADMETLab webserver. Here, the designed molecules appear chronologically from VASP-01 to VASP-06.

VASP-01

Physicochemical Property

Property	Predicted values	Suggestions	Meaning & Preference	Reference
LogS (Solubility)	-5.701 log mol/L (0.831 μg/mL)	> 10 μg/ml	• Optimal: higher than -4 log mol/L<br>• <10 μg/mL: Low solubility; 10–60 μg/mL: Moderate solubility; >60 μg/mL: High solubility	• Book: ISBN: 9787562832287. pp. 14<br>• J PHARMACOL TOX MET. 2000, 44 (1), 235−249;
$LogD_{7.4}$ (Distribution Coefficient D)	2.845	1~5	• < 1: Solubility high; Permeability low by passive transcellular diffusion; Permeability possible via paracellular if MW < 200; Metabolism low.<br>• 1 to 3: Solubility moderate; Permeability moderate; Metabolism low.<br>• 3 to 5: Solubility low; Permeability high; Metabolism moderate to high.<br>• > 5: Solubility low; Permeability high; Metabolism high.	• Methods and principles in medicinal chemistry 18 (pp. 21–45). Weinheim: Wiley-VCH.
LogP (Distribution Coefficient P)	4.336	0~3	• Optimal: 0< LogP <3<br>• LogP <0: poor lipid bilayer permeability.<br>• LogP >3: poor aqueous solubility.	• Book: ISBN: 3-906390-22-5. pp. 127–182.

Absorption

Property	Predicted values	Probability	Suggestions	Meaning & Preference	Reference
Papp (Caco-2 Permeability)	-4.935 cm/s		> -5.15 cm/s	Optimal: higher than -5.15 Log unit or -4.70 or -4.80	• J CHEM INF MODEL. 2016, 56 (4), pp 763–773.
Pgp-inhibitor	-	0.38		• The Pgp-inhibitor & non-inhibitor classification criteria refers the reference.	• J CHEM INF MODEL. 2010. 50(6): p. 1034-1041.<br>• J MED CHEM. 2011. 54(6): p. 1740-1751.
Pgp-substrate	---	0.041		• More likely to be a Pgp substrate: N+O ≥ 8; MW > 400; Acid with pKa > 4<br>• More likely to be a Pgp non-substrate: N+O ≤ 4; MW < 400; Acid with pKa < 8	• J DRUG TARGET. 11, 391–406.
HIA (Human Intestinal Absorption)	++	0.874		• ≥30%: HIA+; <30%: HIA-	• RSC ADV. 2017, 7, 19007-19018

Property	Predicted values	Probability	Suggestions	Meaning & Preference	Reference
F (20% Bioavailability)	++	0.731		• ≥20%: F20+; <20%: F20-	• MOL PHARMACEUT, 2011. 8(3): p. 841-851<br>• J PHARMACEUT BIOMED, 2008. 47(4): p. 677-682.
F (30% Bioavailability)	+	0.578		• ≥30%: F30+; <30%: F30-	• MOL PHARMACEUT, 2011. 8(3): p. 841-851<br>• J PHARMACEUT BIOMED, 2008. 47(4): p. 677-682.

Distribution

Property	Predicted values	Probability	Suggestions	Meaning & Preference	Reference
PPB (Plasma Protein Binding)	89.239 %		90%	• Significant with drugs that are highly protein-bound and have a low therapeutic index.	• ISBN: 978-0-1236-9520-8. pp. 194
VD (Volume Distribution)	0.084 L/kg		0.04~20 L/kg	• Optimal: 0.04-20L/kg;<br>• Range:<br><0.07L/kg: Confined to blood, Bound to plasma protein or highly hydrophilic; 0.07-0.7L/kg: Evenly distributed; >0.7L/kg: Bound to tissue components (e.g., protein, lipid),highly lipophilic.	• Book: ISBN: 9787562832287. pp. 174<br>• Book: ISBN: 978-0-1236-9520-8. pp. 229
BBB (Blood–Brain Barrier)	++	0.861		• BB ratio >=0.1: BBB+; BB ratio <0.1: BBB-<br>• These features tend to improve BBB permeation:<br>H-bonds (total) < 8–10; MW < 400–500; No acids.	• J NEUROCHEM. 70, 1781–1792

Metabolism

Property	Predicted values	Probability	Meaning & Preference	Reference
P450 CYP1A2 inhibitor	++	0.863	• Molecules that labeled inhibitor in PubChem BioAssay were regarded as inhibitor.	• NAT BIOTECHNOL. 2009, 27(11): 1050-1055.<br>• BIOINFORMATICS. 2013, 29(16): 2051-2052.

Property	Predicted values	Probability	Meaning & Preference	Reference
P450 CYP1A2 Substrate	+	0.694	• Molecules that labeled substrate in PubChem BioAssay were regarded as substrate.<br>• Characteristics of CYP1A2 substrate: 0.08< LogP <3.61; Planar amines and amides	• NAT BIOTECHNOL. 2009, 27(11): 1050-1055.<br>• BIOINFORMATICS. 2013, 29(16): 2051-2052.
P450 CYP3A4 inhibitor	+++	0.964	• Molecules that labeled inhibitor in PubChem BioAssay were regarded as inhibitor.<br>• Strategies to Reduce CYP3A4 Inhibition: Decrease the lipophilicity (LogD $_{7.4}$); Add steric hindrance to the heterocycle para to the nitrogen; Add an electronic substitution (e.g., halogen) that reduces the pKa of the nitrogen.	• NAT BIOTECHNOL. 2009, 27(11): 1050-1055.<br>• BIOINFORMATICS. 2013, 29(16): 2051-2052.
P450 CYP3A4 substrate	-	0.424	• Molecules that labeled substrate in PubChem BioAssay were regarded as substrate.<br>• Characteristics of CYP3A4 substrate: 0.97< LogP <7.54; Large molecules	• NAT BIOTECHNOL. 2009, 27(11): 1050-1055.<br>• BIOINFORMATICS. 2013, 29(16): 2051-2052.<br>• ISBN: 978-0-1236-9520-8. pp. 162
P450 CYP2C9 inhibitor	+	0.509	• Molecules that labeled inhibitor in PubChem BioAssay were regarded as inhibitor.	• NAT BIOTECHNOL. 2009, 27(11): 1050-1055.<br>• BIOINFORMATICS. 2013, 29(16): 2051-2052.
P450 CYP2C9 substrate	-	0.406	• Molecules that labeled substrate in PubChem BioAssay were regarded as substrate.<br>• Characteristics of CYP2C9 substrate: 0.89< LogP <5.18; Acidic (Nonionized)	• MOL INFORM. 2011. 30(10): p. 885-895.<br>• J CHEM INF MODEL. 2013. 53(12): p. 3373-3383.<br>• ISBN: 978-0-1236-9520-8. pp. 162
P450 CYP2C19 inhibitor	+	0.583	• Molecules that labeled inhibitor in PubChem BioAssay were regarded as inhibitor.	• NAT BIOTECHNOL. 2009, 27(11): 1050-1055.<br>• BIOINFORMATICS. 2013, 29(16): 2051-2052.

Property	Predicted values	Probability	Meaning & Preference	Reference
P450 CYP2C19 substrate	-	0.408	• Molecules that labeled substrate in PubChem BioAssay were regarded as substrate.	• NAT BIOTECHNOL. 2009, 27(11): 1050-1055.<br>• BIOINFORMATICS. 2013, 29(16): 2051-2052.
P450 CYP2D6 inhibitor	+	0.551	• Molecules that labeled inhibitor in PubChem BioAssay were regarded as inhibitor.	• MOL INFORM. 2011. 30(10): p. 885-895.<br>• J CHEM INF MODEL. 2013. 53(12): p. 3373-3383.
P450 CYP2D6 substrate	-	0.351	• Molecules that labeled substrate in PubChem BioAssay were regarded as substrate.<br>• Characteristics of CYP2D6 substrate: 0.75< LogP <5.04; Basic (Ionized)	• MOL INFORM. 2011. 30(10): p. 885-895.<br>• J CHEM INF MODEL. 2013. 53(12): p. 3373-3383.<br>• ISBN: 978-0-1236-9520-8. pp. 162

Elimination

Property	Predicted values	Suggestions	Meaning & Preference	Reference
$T_{1/2}$ (Half Life Time)	2.008 h	> 0.5 h	• Range: >8h: high; 3h< Cl < 8h: moderate; <3h: low	• ISBN: 978-0-1236-9520-8. pp. 236
CL (Clearance Rate)	0.951 mL/min/kg		• Range: >15 mL/min/kg: high; 5mL/min/kg< Cl < 15mL/min/kg: moderate; <5 mL/min/kg: low	• ISBN: 978-0-1236-9520-8. pp. 236

Toxicity

Property	Predicted values	Probability	Suggestions	Meaning & Preference	Reference
hERG (hERG Blockers)	++	0.859		• Where molecules with IC50 < 40 μM were regarded as blockers.<br>• Features may lead to hERG blocker:<br>○ A basic amine (positively ionizable, pKa >7.3).<br>○ Hydrophobic/lipophilic substructure(s) (ClogP >3.7).<br>○ Absence of negatively ionizable groups or oxygen H-bond acceptors.	• TRENDS PHARMACOL SCI. 2005, 26(3): 119-124<br>• ISBN: 978-0-1236-9520-8. pp. 213<br>• MOL PHARM. 2016, 13(8):2855–2866
H-HT (Human Hepatotoxicity)	+	0.622		• The H-HT positive(+) & negative(-) classification criteria refers the reference.	• CHEM RES TOXICOL, 2016, 29(5): 757-767.
AMES (Ames Mutagenicity)	-	0.468		• Ames positive(+) & negative(-): significantly induces revertant colony growth at least in one out of usually five strains, otherwise, negative.	• J CHEM INF MODEL. 2012, 52(11): 2840-2847.
SkinSen (Skin sensitization)	-	0.487		• Sensitizer & Non-sensitizer: The (r)LLNA experimental value. (r)LLNA: (Reduced) local lymph node assay.	• TOXICOL APPL PHARM, 2015 , 284 (2) :262-272
LD50 (LD50 of acute toxicity)	2.565 -log mol/kg (1137.237 mg/kg)		> 500 mg/kg	• Median lethal dose (LD50) usually represents the acute toxicity of chemicals.It is the dose amount of a tested molecule to kill 50 % of the treated animals within a given period.<br>• High-toxicity: 1~50 mg/kg; Toxicity: 51~500 mg/kg; low-toxicity: 501~5000 mg/kg.	• CHEM RES TOXICOL, 2009, 22 (12), pp 1913–1921<br>• J CHEMINFORMATICS, 2016 , 8 (1) :6

Property	Predicted values	Probability	Suggestions	Meaning & Preference	Reference
DILI (Drug Induced Liver Injury)	+	0.698		• The DILI positive(+) & negative(-) classification criteria refers the reference.	• J CHEM INF MODEL, 2015, 55(10) :2085-2093

VASP-02

Physicochemical Property

Property	Predicted values	Suggestions	Meaning & Preference	Reference
LogS (Solubility)	-5.02 log mol/L (3.517 μg/mL)	> 10 μg/ml	• Optimal: higher than -4 log mol/L<br>• <10 μg/mL: Low solubility; 10–60 μg/mL: Moderate solubility; >60 μg/mL: High solubility	• Book: ISBN: 9787562832287. pp. 14<br>• J PHARMACOL TOX MET. 2000, 44 (1), 235−249;
$LogD_{7.4}$ (Distribution Coefficient D)	2.538	1~5	• < 1: Solubility high; Permeability low by passive transcellular diffusion; Permeability possible via paracellular if MW < 200; Metabolism low.<br>• 1 to 3: Solubility moderate; Permeability moderate; Metabolism low.<br>• 3 to 5: Solubility low; Permeability high; Metabolism moderate to high.<br>• > 5: Solubility low; Permeability high; Metabolism high.	• Methods and principles in medicinal chemistry 18 (pp. 21–45). Weinheim: Wiley-VCH.
LogP (Distribution Coefficient P)	3.528	0~3	• Optimal: 0< LogP <3<br>• LogP <0: poor lipid bilayer permeability.<br>• LogP >3: poor aqueous solubility.	• Book: ISBN: 3-906390-22-5. pp. 127–182.

Absorption

Property	Predicted values	Probability	Suggestions	Meaning & Preference	Reference
Papp (Caco-2 Permeability)	-4.855 cm/s		> -5.15 cm/s	Optimal: higher than -5.15 Log unit or -4.70 or -4.80	• J CHEM INF MODEL. 2016, 56 (4), pp 763–773.
Pgp-inhibitor	-	0.492		• The Pgp-inhibitor & non-inhibitor classification criteria refers the reference.	• J CHEM INF MODEL. 2010. 50(6): p. 1034-1041.<br>• J MED CHEM. 2011. 54(6): p. 1740-1751.
Pgp-substrate	---	0.05		• More likely to be a Pgp substrate: N+O ≥ 8; MW > 400; Acid with pKa > 4<br>• More likely to be a Pgp non-substrate: N+O ≤ 4; MW < 400; Acid with pKa < 8	• J DRUG TARGET. 11, 391–406.
HIA (Human Intestinal Absorption)	++	0.877		• ≥30%: HIA+; <30%: HIA-	• RSC ADV. 2017, 7, 19007-19018

Property	Predicted values	Probability	Suggestions	Meaning & Preference	Reference
F (20% Bioavailability)	++	0.77		• ≥20%: F20+; <20%: F20-	• MOL PHARMACEUT, 2011. 8(3): p. 841-851<br>• J PHARMACEUT BIOMED, 2008. 47(4): p. 677-682.
F (30% Bioavailability)	+	0.6		• ≥30%: F30+; <30%: F30-	• MOL PHARMACEUT, 2011. 8(3): p. 841-851<br>• J PHARMACEUT BIOMED, 2008. 47(4): p. 677-682.

Distribution

Property	Predicted values	Probability	Suggestions	Meaning & Preference	Reference
PPB (Plasma Protein Binding)	85.943 %		90%	• Significant with drugs that are highly protein-bound and have a low therapeutic index.	• ISBN: 978-0-1236-9520-8. pp. 194
VD (Volume Distribution)	-0.139 L/kg		0.04~20 L/kg	• Optimal: 0.04-20L/kg;<br>• Range: <0.07L/kg: Confined to blood, Bound to plasma protein or highly hydrophilic; 0.07-0.7L/kg: Evenly distributed; >0.7L/kg: Bound to tissue components (e.g., protein, lipid),highly lipophilic.	• Book: ISBN: 9787562832287. pp. 174<br>• Book: ISBN: 978-0-1236-9520-8. pp. 229
BBB (Blood–Brain Barrier)	+++	0.907		• BB ratio >=0.1: BBB+; BB ratio <0.1: BBB-<br>• These features tend to improve BBB permeation: H-bonds (total) < 8–10; MW < 400–500; No acids.	• J NEUROCHEM. 70, 1781–1792

Metabolism

Property	Predicted values	Probability	Meaning & Preference	Reference
P450 CYP1A2 inhibitor	+++	0.9	• Molecules that labeled inhibitor in PubChem BioAssay were regarded as inhibitor.	• NAT BIOTECHNOL. 2009, 27(11): 1050-1055.<br>• BIOINFORMATICS. 2013, 29(16): 2051-2052.

Property	Predicted values	Probability	Meaning & Preference	Reference
P450 CYP1A2 Substrate	++	0.736	• Molecules that labeled substrate in PubChem BioAssay were regarded as substrate.<br>• Characteristics of CYP1A2 substrate: 0.08< LogP <3.61; Planar amines and amides	• NAT BIOTECHNOL. 2009, 27(11): 1050-1055.<br>• BIOINFORMATICS. 2013, 29(16): 2051-2052.
P450 CYP3A4 inhibitor	+++	0.955	• Molecules that labeled inhibitor in PubChem BioAssay were regarded as inhibitor.<br>• Strategies to Reduce CYP3A4 Inhibition: Decrease the lipophilicity (LogD $_{7.4}$); Add steric hindrance to the heterocycle para to the nitrogen; Add an electronic substitution (e.g., halogen) that reduces the pKa of the nitrogen.	• NAT BIOTECHNOL. 2009, 27(11): 1050-1055.<br>• BIOINFORMATICS. 2013, 29(16): 2051-2052.
P450 CYP3A4 substrate	-	0.416	• Molecules that labeled substrate in PubChem BioAssay were regarded as substrate.<br>• Characteristics of CYP3A4 substrate: 0.97< LogP <7.54; Large molecules	• NAT BIOTECHNOL. 2009, 27(11): 1050-1055.<br>• BIOINFORMATICS. 2013, 29(16): 2051-2052.<br>• ISBN: 978-0-1236-9520-8. pp. 162
P450 CYP2C9 inhibitor	-	0.368	• Molecules that labeled inhibitor in PubChem BioAssay were regarded as inhibitor.	• NAT BIOTECHNOL. 2009, 27(11): 1050-1055.<br>• BIOINFORMATICS. 2013, 29(16): 2051-2052.
P450 CYP2C9 substrate	-	0.393	• Molecules that labeled substrate in PubChem BioAssay were regarded as substrate.<br>• Characteristics of CYP2C9 substrate: 0.89< LogP <5.18; Acidic (Nonionized)	• MOL INFORM. 2011. 30(10): p. 885-895.<br>• J CHEM INF MODEL. 2013. 53(12): p. 3373-3383.<br>• ISBN: 978-0-1236-9520-8. pp. 162
P450 CYP2C19 inhibitor	+	0.583	• Molecules that labeled inhibitor in PubChem BioAssay were regarded as inhibitor.	• NAT BIOTECHNOL. 2009, 27(11): 1050-1055.<br>• BIOINFORMATICS. 2013, 29(16): 2051-2052.

Property	Predicted values	Probability	Meaning & Preference	Reference
P450 CYP2C19 substrate	-	0.372	• Molecules that labeled substrate in PubChem BioAssay were regarded as substrate.	• NAT BIOTECHNOL. 2009, 27(11): 1050-1055.<br>• BIOINFORMATICS. 2013, 29(16): 2051-2052.
P450 CYP2D6 inhibitor	+	0.56	• Molecules that labeled inhibitor in PubChem BioAssay were regarded as inhibitor.	• MOL INFORM. 2011. 30(10): p. 885-895.<br>• J CHEM INF MODEL. 2013. 53(12): p. 3373-3383.
P450 CYP2D6 substrate	-	0.382	• Molecules that labeled substrate in PubChem BioAssay were regarded as substrate.<br>• Characteristics of CYP2D6 substrate: 0.75< LogP <5.04; Basic (Ionized)	• MOL INFORM. 2011. 30(10): p. 885-895.<br>• J CHEM INF MODEL. 2013. 53(12): p. 3373-3383.<br>• ISBN: 978-0-1236-9520-8. pp. 162

Elimination

Property	Predicted values	Suggestions	Meaning & Preference	Reference
$T_{1/2}$ (Half Life Time)	1.658 h	> 0.5 h	• Range: >8h: high; 3h< Cl < 8h: moderate; <3h: low	• ISBN: 978-0-1236-9520-8. pp. 236
CL (Clearance Rate)	1.352 mL/min/kg		• Range: >15 mL/min/kg: high; 5mL/min/kg< Cl < 15mL/min/kg: moderate; <5 mL/min/kg: low	• ISBN: 978-0-1236-9520-8. pp. 236

Toxicity

Property	Predicted values	Probability	Suggestions	Meaning & Preference	Reference
hERG (hERG Blockers)	++	0.841		• Where molecules with IC50 < 40 μM were regarded as blockers.<br>• Features may lead to hERG blocker:<br>○ A basic amine (positively ionizable, pKa >7.3).<br>○ Hydrophobic/lipophilic substructure(s) (ClogP >3.7).<br>○ Absence of negatively ionizable groups or oxygen H-bond acceptors.	• TRENDS PHARMACOL SCI. 2005, 26(3): 119-124<br>• ISBN: 978-0-1236-9520-8. pp. 213<br>• MOL PHARM. 2016, 13(8):2855–2866
H-HT (Human Hepatotoxicity)	+	0.54		• The H-HT positive(+) & negative(-) classification criteria refers the reference.	• CHEM RES TOXICOL, 2016, 29(5): 757-767.
AMES (Ames Mutagenicity)	-	0.472		• Ames positive(+) & negative(-): significantly induces revertant colony growth at least in one out of usually five strains, otherwise, negative.	• J CHEM INF MODEL. 2012, 52(11): 2840-2847.
SkinSen (Skin sensitization)	-	0.414		• Sensitizer & Non-sensitizer: The (r)LLNA experimental value. (r)LLNA: (Reduced) local lymph node assay.	• TOXICOL APPL PHARM, 2015 , 284 (2) :262-272
LD50 (LD50 of acute toxicity)	2.515 -log mol/kg (1125.195 mg/kg)		> 500 mg/kg	• Median lethal dose (LD50) usually represents the acute toxicity of chemicals.It is the dose amount of a tested molecule to kill 50 % of the treated animals within a given period.<br>• High-toxicity: 1~50 mg/kg; Toxicity: 51~500 mg/kg; low-toxicity: 501~5000 mg/kg.	• CHEM RES TOXICOL, 2009, 22 (12), pp 1913–1921<br>• J CHEMINFORMATICS, 2016 , 8 (1) :6

Property	Predicted values	Probability	Suggestions	Meaning & Preference	Reference
DILI (Drug Induced Liver Injury)	+	0.696		• The DILI positive(+) & negative(-) classification criteria refers the reference.	• J CHEM INF MODEL, 2015, 55(10) :2085-2093

VASP-03

Physicochemical Property

Property	Predicted values	Suggestions	Meaning & Preference	Reference
LogS (Solubility)	-4.867 log mol/L (5.22 μg/mL)	> 10 μg/ml	• Optimal: higher than -4 log mol/L<br>• <10 μg/mL: Low solubility; 10–60 μg/mL: Moderate solubility; >60 μg/mL: High solubility	• Book: ISBN: 9787562832287. pp. 14<br>• J PHARMACOL TOX MET. 2000, 44 (1), 235−249;
$LogD_{7.4}$ (Distribution Coefficient D)	2.585	1~5	• < 1: Solubility high; Permeability low by passive transcellular diffusion; Permeability possible via paracellular if MW < 200; Metabolism low.<br>• 1 to 3: Solubility moderate; Permeability moderate; Metabolism low.<br>• 3 to 5: Solubility low; Permeability high; Metabolism moderate to high.<br>• > 5: Solubility low; Permeability high; Metabolism high.	• Methods and principles in medicinal chemistry 18 (pp. 21–45). Weinheim: Wiley-VCH.
LogP (Distribution Coefficient P)	3.407	0~3	• Optimal: 0< LogP <3<br>• LogP <0: poor lipid bilayer permeability.<br>• LogP >3: poor aqueous solubility.	• Book: ISBN: 3-906390-22-5. pp. 127–182.

Absorption

Property	Predicted values	Probability	Suggestions	Meaning & Preference	Reference
Papp (Caco-2 Permeability)	-4.827 cm/s		> -5.15 cm/s	Optimal: higher than -5.15 Log unit or -4.70 or -4.80	• J CHEM INF MODEL. 2016, 56 (4), pp 763–773.
Pgp-inhibitor	+	0.671		• The Pgp-inhibitor & non-inhibitor classification criteria refers the reference.	• J CHEM INF MODEL. 2010. 50(6): p. 1034-1041.<br>• J MED CHEM. 2011. 54(6): p. 1740-1751.
Pgp-substrate	---	0.016		• More likely to be a Pgp substrate: N+O ≥ 8; MW > 400; Acid with pKa > 4<br>• More likely to be a Pgp non-substrate: N+O ≤ 4; MW < 400; Acid with pKa < 8	• J DRUG TARGET. 11, 391–406.
HIA (Human Intestinal Absorption)	++	0.775		• ≥30%: HIA+; <30%: HIA-	• RSC ADV. 2017, 7, 19007-19018

Property	Predicted values	Probability	Suggestions	Meaning & Preference	Reference
F (20% Bioavailability)	++	0.761		• ≥20%: F20+; <20%: F20-	• MOL PHARMACEUT, 2011. 8(3): p. 841-851<br>• J PHARMACEUT BIOMED, 2008. 47(4): p. 677-682.
F (30% Bioavailability)	+	0.589		• ≥30%: F30+; <30%: F30-	• MOL PHARMACEUT, 2011. 8(3): p. 841-851<br>• J PHARMACEUT BIOMED, 2008. 47(4): p. 677-682.

Distribution

Property	Predicted values	Probability	Suggestions	Meaning & Preference	Reference
PPB (Plasma Protein Binding)	85.791 %		90%	• Significant with drugs that are highly protein-bound and have a low therapeutic index.	• ISBN: 978-0-1236-9520-8. pp. 194
VD (Volume Distribution)	-0.404 L/kg		0.04~20 L/kg	• Optimal: 0.04-20L/kg;<br>• Range: <0.07L/kg: Confined to blood, Bound to plasma protein or highly hydrophilic; 0.07-0.7L/kg: Evenly distributed; >0.7L/kg: Bound to tissue components (e.g., protein, lipid),highly lipophilic.	• Book: ISBN: 9787562832287. pp. 174<br>• Book: ISBN: 978-0-1236-9520-8. pp. 229
BBB (Blood–Brain Barrier)	+++	0.923		• BB ratio >=0.1: BBB+; BB ratio <0.1: BBB-<br>• These features tend to improve BBB permeation: H-bonds (total) < 8–10; MW < 400–500; No acids.	• J NEUROCHEM. 70, 1781–1792

Metabolism

Property	Predicted values	Probability	Meaning & Preference	Reference
P450 CYP1A2 inhibitor	+++	0.9	• Molecules that labeled inhibitor in PubChem BioAssay were regarded as inhibitor.	• NAT BIOTECHNOL. 2009, 27(11): 1050-1055.<br>• BIOINFORMATICS. 2013, 29(16): 2051-2052.

Property	Predicted values	Probability	Meaning & Preference	Reference
P450 CYP1A2 Substrate	++	0.768	• Molecules that labeled substrate in PubChem BioAssay were regarded as substrate.<br>• Characteristics of CYP1A2 substrate: 0.08< LogP <3.61; Planar amines and amides	• NAT BIOTECHNOL. 2009, 27(11): 1050-1055.<br>• BIOINFORMATICS. 2013, 29(16): 2051-2052.
P450 CYP3A4 inhibitor	+++	0.918	• Molecules that labeled inhibitor in PubChem BioAssay were regarded as inhibitor.<br>• Strategies to Reduce CYP3A4 Inhibition: Decrease the lipophilicity ($LogD_{7.4}$); Add steric hindrance to the heterocycle para to the nitrogen; Add an electronic substitution (e.g., halogen) that reduces the pKa of the nitrogen.	• NAT BIOTECHNOL. 2009, 27(11): 1050-1055.<br>• BIOINFORMATICS. 2013, 29(16): 2051-2052.
P450 CYP3A4 substrate	-	0.426	• Molecules that labeled substrate in PubChem BioAssay were regarded as substrate.<br>• Characteristics of CYP3A4 substrate: 0.97< LogP <7.54; Large molecules	• NAT BIOTECHNOL. 2009, 27(11): 1050-1055.<br>• BIOINFORMATICS. 2013, 29(16): 2051-2052.<br>• ISBN: 978-0-1236-9520-8. pp. 162
P450 CYP2C9 inhibitor	+	0.578	• Molecules that labeled inhibitor in PubChem BioAssay were regarded as inhibitor.	• NAT BIOTECHNOL. 2009, 27(11): 1050-1055.<br>• BIOINFORMATICS. 2013, 29(16): 2051-2052.
P450 CYP2C9 substrate	-	0.388	• Molecules that labeled substrate in PubChem BioAssay were regarded as substrate.<br>• Characteristics of CYP2C9 substrate: 0.89< LogP <5.18; Acidic (Nonionized)	• MOL INFORM. 2011. 30(10): p. 885-895.<br>• J CHEM INF MODEL. 2013. 53(12): p. 3373-3383.<br>• ISBN: 978-0-1236-9520-8. pp. 162
P450 CYP2C19 inhibitor	++	0.714	• Molecules that labeled inhibitor in PubChem BioAssay were regarded as inhibitor.	• NAT BIOTECHNOL. 2009, 27(11): 1050-1055.<br>• BIOINFORMATICS. 2013, 29(16): 2051-2052.

Property	Predicted values	Probability	Meaning & Preference	Reference
P450 CYP2C19 substrate	-	0.378	• Molecules that labeled substrate in PubChem BioAssay were regarded as substrate.	• NAT BIOTECHNOL. 2009, 27(11): 1050-1055.<br>• BIOINFORMATICS. 2013, 29(16): 2051-2052.
P450 CYP2D6 inhibitor	+	0.554	• Molecules that labeled inhibitor in PubChem BioAssay were regarded as inhibitor.	• MOL INFORM. 2011. 30(10): p. 885-895.<br>• J CHEM INF MODEL. 2013. 53(12): p. 3373-3383.
P450 CYP2D6 substrate	-	0.364	• Molecules that labeled substrate in PubChem BioAssay were regarded as substrate.<br>• Characteristics of CYP2D6 substrate: 0.75< LogP <5.04; Basic (Ionized)	• MOL INFORM. 2011. 30(10): p. 885-895.<br>• J CHEM INF MODEL. 2013. 53(12): p. 3373-3383.<br>• ISBN: 978-0-1236-9520-8. pp. 162

Elimination

Property	Predicted values	Suggestions	Meaning & Preference	Reference
$T_{1/2}$ (Half Life Time)	1.651 h	> 0.5 h	• Range: >8h: high; 3h< Cl < 8h: moderate; <3h: low	• ISBN: 978-0-1236-9520-8. pp. 236
CL (Clearance Rate)	1.609 mL/min/kg		• Range: >15 mL/min/kg: high; 5mL/min/kg< Cl < 15mL/min/kg: moderate; <5 mL/min/kg: low	• ISBN: 978-0-1236-9520-8. pp. 236

Toxicity

Property	Predicted values	Probability	Suggestions	Meaning & Preference	Reference
hERG (hERG Blockers)	++	0.775		• Where molecules with IC50 < 40 μM were regarded as blockers.<br>• Features may lead to hERG blocker:<br>o A basic amine (positively ionizable, pKa >7.3).<br>o Hydrophobic/lipophilic substructure(s) (ClogP >3.7).<br>o Absence of negatively ionizable groups or oxygen H-bond acceptors.	• TRENDS PHARMACOL SCI. 2005, 26(3): 119-124<br>• ISBN: 978-0-1236-9520-8. pp. 213<br>• MOL PHARM. 2016, 13(8):2855–2866
H-HT (Human Hepatotoxicity)	-	0.452		• The H-HT positive(+) & negative(-) classification criteria refers the reference.	• CHEM RES TOXICOL, 2016, 29(5): 757-767.
AMES (Ames Mutagenicity)	-	0.386		• Ames positive(+) & negative(-): significantly induces revertant colony growth at least in one out of usually five strains, otherwise, negative.	• J CHEM INF MODEL. 2012, 52(11): 2840-2847.
SkinSen (Skin sensitization)	-	0.321		• Sensitizer & Non-sensitizer: The (r)LLNA experimental value. (r)LLNA: (Reduced) local lymph node assay.	• TOXICOL APPL PHARM, 2015 , 284 (2) :262-272
LD50 (LD50 of acute toxicity)	3.046 -log mol/kg (345.696 mg/kg)		> 500 mg/kg	• Median lethal dose (LD50) usually represents the acute toxicity of chemicals.It is the dose amount of a tested molecule to kill 50 % of the treated animals within a given period.<br>• High-toxicity: 1~50 mg/kg; Toxicity: 51~500 mg/kg; low-toxicity: 501~5000 mg/kg.	• CHEM RES TOXICOL, 2009, 22 (12), pp 1913–1921<br>• J CHEMINFORMATICS, 2016 , 8 (1) :6

Property	Predicted values	Probability	Suggestions	Meaning & Preference	Reference
DILI (Drug Induced Liver Injury)	++	0.75		• The DILI positive(+) & negative(-) classification criteria refers the reference.	• J CHEM INF MODEL, 2015, 55(10) :2085-2093

VASP-04

Physicochemical Property

Property	Predicted values	Suggestions	Meaning & Preference	Reference
LogS (Solubility)	-5.409 log mol/L (1.719 μg/mL)	> 10 μg/ml	• Optimal: higher than -4 log mol/L<br>• <10 μg/mL: Low solubility; 10–60 μg/mL: Moderate solubility; >60 μg/mL: High solubility	• Book: ISBN: 9787562832287. pp. 14<br>• J PHARMACOL TOX MET. 2000, 44 (1), 235−249;
$LogD_{7.4}$ (Distribution Coefficient D)	2.801	1~5	• < 1: Solubility high; Permeability low by passive transcellular diffusion; Permeability possible via paracellular if MW < 200; Metabolism low.<br>• 1 to 3: Solubility moderate; Permeability moderate; Metabolism low.<br>• 3 to 5: Solubility low; Permeability high; Metabolism moderate to high.<br>• > 5: Solubility low; Permeability high; Metabolism high.	• Methods and principles in medicinal chemistry 18 (pp. 21–45). Weinheim: Wiley-VCH.
LogP (Distribution Coefficient P)	4.884	0~3	• Optimal: 0< LogP <3<br>• LogP <0: poor lipid bilayer permeability.<br>• LogP >3: poor aqueous solubility.	• Book: ISBN: 3-906390-22-5. pp. 127–182.

Absorption

Property	Predicted values	Probability	Suggestions	Meaning & Preference	Reference
Papp (Caco-2 Permeability)	-4.67 cm/s		> -5.15 cm/s	Optimal: higher than -5.15 Log unit or -4.70 or -4.80	• J CHEM INF MODEL. 2016, 56 (4), pp 763–773.
Pgp-inhibitor	+	0.642		• The Pgp-inhibitor & non-inhibitor classification criteria refers the reference.	• J CHEM INF MODEL. 2010. 50(6): p. 1034-1041.<br>• J MED CHEM. 2011. 54(6): p. 1740-1751.
Pgp-substrate	---	0.038		• More likely to be a Pgp substrate: N+O ≥ 8; MW > 400; Acid with pKa > 4<br>• More likely to be a Pgp non-substrate: N+O ≤ 4; MW < 400; Acid with pKa < 8	• J DRUG TARGET. 11, 391–406.
HIA (Human Intestinal Absorption)	++	0.779		• ≥30%: HIA+; <30%: HIA-	• RSC ADV. 2017, 7, 19007-19018

Property	Predicted values	Probability	Suggestions	Meaning & Preference	Reference
F (20% Bioavailability)	+	0.631		• ≥20%: F20+; <20%: F20-	• MOL PHARMACEUT, 2011. 8(3): p. 841-851<br>• J PHARMACEUT BIOMED, 2008. 47(4): p. 677-682.
F (30% Bioavailability)	+	0.551		• ≥30%: F30+; <30%: F30-	• MOL PHARMACEUT, 2011. 8(3): p. 841-851<br>• J PHARMACEUT BIOMED, 2008. 47(4): p. 677-682.

Distribution

Property	Predicted values	Probability	Suggestions	Meaning & Preference	Reference
PPB (Plasma Protein Binding)	91.183 %		90%	• Significant with drugs that are highly protein-bound and have a low therapeutic index.	• ISBN: 978-0-1236-9520-8. pp. 194
VD (Volume Distribution)	0.127 L/kg		0.04~20 L/kg	• Optimal: 0.04-20L/kg;<br>• Range: <0.07L/kg: Confined to blood, Bound to plasma protein or highly hydrophilic; 0.07-0.7L/kg: Evenly distributed; >0.7L/kg: Bound to tissue components (e.g., protein, lipid),highly lipophilic.	• Book: ISBN: 9787562832287. pp. 174<br>• Book: ISBN: 978-0-1236-9520-8. pp. 229
BBB (Blood–Brain Barrier)	++	0.808		• BB ratio >=0.1: BBB+; BB ratio <0.1: BBB-<br>• These features tend to improve BBB permeation: H-bonds (total) < 8–10; MW < 400–500; No acids.	• J NEUROCHEM. 70, 1781–1792

Metabolism

Property	Predicted values	Probability	Meaning & Preference	Reference
P450 CYP1A2 inhibitor	+	0.587	• Molecules that labeled inhibitor in PubChem BioAssay were regarded as inhibitor.	• NAT BIOTECHNOL. 2009, 27(11): 1050-1055.<br>• BIOINFORMATICS. 2013, 29(16): 2051-2052.

Property	Predicted values	Probability	Meaning & Preference	Reference
P450 CYP1A2 Substrate	+	0.516	• Molecules that labeled substrate in PubChem BioAssay were regarded as substrate.<br>• Characteristics of CYP1A2 substrate: 0.08< LogP <3.61; Planar amines and amides	• NAT BIOTECHNOL. 2009, 27(11): 1050-1055.<br>• BIOINFORMATICS. 2013, 29(16): 2051-2052.
P450 CYP3A4 inhibitor	++	0.71	• Molecules that labeled inhibitor in PubChem BioAssay were regarded as inhibitor.<br>• Strategies to Reduce CYP3A4 Inhibition: Decrease the lipophilicity ($LogD_{7.4}$); Add steric hindrance to the heterocycle para to the nitrogen; Add an electronic substitution (e.g., halogen) that reduces the pKa of the nitrogen.	• NAT BIOTECHNOL. 2009, 27(11): 1050-1055.<br>• BIOINFORMATICS. 2013, 29(16): 2051-2052.
P450 CYP3A4 substrate	-	0.438	• Molecules that labeled substrate in PubChem BioAssay were regarded as substrate.<br>• Characteristics of CYP3A4 substrate: 0.97< LogP <7.54; Large molecules	• NAT BIOTECHNOL. 2009, 27(11): 1050-1055.<br>• BIOINFORMATICS. 2013, 29(16): 2051-2052.<br>• ISBN: 978-0-1236-9520-8. pp. 162
P450 CYP2C9 inhibitor	-	0.472	• Molecules that labeled inhibitor in PubChem BioAssay were regarded as inhibitor.	• NAT BIOTECHNOL. 2009, 27(11): 1050-1055.<br>• BIOINFORMATICS. 2013, 29(16): 2051-2052.
P450 CYP2C9 substrate	-	0.365	• Molecules that labeled substrate in PubChem BioAssay were regarded as substrate.<br>• Characteristics of CYP2C9 substrate: 0.89< LogP <5.18; Acidic (Nonionized)	• MOL INFORM. 2011. 30(10): p. 885-895.<br>• J CHEM INF MODEL. 2013. 53(12): p. 3373-3383.<br>• ISBN: 978-0-1236-9520-8. pp. 162
P450 CYP2C19 inhibitor	++	0.776	• Molecules that labeled inhibitor in PubChem BioAssay were regarded as inhibitor.	• NAT BIOTECHNOL. 2009, 27(11): 1050-1055.<br>• BIOINFORMATICS. 2013, 29(16): 2051-2052.

Property	Predicted values	Probability	Meaning & Preference	Reference
P450 CYP2C19 substrate	-	0.387	• Molecules that labeled substrate in PubChem BioAssay were regarded as substrate.	• NAT BIOTECHNOL. 2009, 27(11): 1050-1055.<br>• BIOINFORMATICS. 2013, 29(16): 2051-2052.
P450 CYP2D6 inhibitor	-	0.394	• Molecules that labeled inhibitor in PubChem BioAssay were regarded as inhibitor.	• MOL INFORM. 2011. 30(10): p. 885-895.<br>• J CHEM INF MODEL. 2013. 53(12): p. 3373-3383.
P450 CYP2D6 substrate	-	0.323	• Molecules that labeled substrate in PubChem BioAssay were regarded as substrate.<br>• Characteristics of CYP2D6 substrate: 0.75< LogP <5.04; Basic (Ionized)	• MOL INFORM. 2011. 30(10): p. 885-895.<br>• J CHEM INF MODEL. 2013. 53(12): p. 3373-3383.<br>• ISBN: 978-0-1236-9520-8. pp. 162

Elimination

Property	Predicted values	Suggestions	Meaning & Preference	Reference
$T_{1/2}$ (Half Life Time)	1.853 h	> 0.5 h	• Range: >8h: high; 3h< Cl < 8h: moderate; <3h: low	• ISBN: 978-0-1236-9520-8. pp. 236
CL (Clearance Rate)	0.981 mL/min/kg		• Range: >15 mL/min/kg: high; 5mL/min/kg< Cl < 15mL/min/kg: moderate; <5 mL/min/kg: low	• ISBN: 978-0-1236-9520-8. pp. 236

Toxicity

Property	Predicted values	Probability	Suggestions	Meaning & Preference	Reference
hERG (hERG Blockers)	+++	0.936		• Where molecules with IC50 < 40 μM were regarded as blockers.<br>• Features may lead to hERG blocker:<br>○ A basic amine (positively ionizable, pKa >7.3).<br>○ Hydrophobic/lipophilic substructure(s) (ClogP >3.7).<br>○ Absence of negatively ionizable groups or oxygen H-bond acceptors.	• TRENDS PHARMACOL SCI. 2005, 26(3): 119-124<br>• ISBN: 978-0-1236-9520-8. pp. 213<br>• MOL PHARM. 2016, 13(8):2855–2866
H-HT (Human Hepatotoxicity)	++	0.716		• The H-HT positive(+) & negative(-) classification criteria refers the reference.	• CHEM RES TOXICOL, 2016, 29(5): 757-767.
AMES (Ames Mutagenicity)	-	0.32		• Ames positive(+) & negative(-): significantly induces revertant colony growth at least in one out of usually five strains, otherwise, negative.	• J CHEM INF MODEL. 2012, 52(11): 2840-2847.
SkinSen (Skin sensitization)	-	0.458		• Sensitizer & Non-sensitizer: The (r)LLNA experimental value. (r)LLNA: (Reduced) local lymph node assay.	• TOXICOL APPL PHARM, 2015 , 284 (2) :262-272
LD50 (LD50 of acute toxicity)	2.441 -log mol/kg (1596.72 mg/kg)		> 500 mg/kg	• Median lethal dose (LD50) usually represents the acute toxicity of chemicals.It is the dose amount of a tested molecule to kill 50 % of the treated animals within a given period.<br>• High-toxicity: 1~50 mg/kg; Toxicity: 51~500 mg/kg; low-toxicity: 501~5000 mg/kg.	• CHEM RES TOXICOL, 2009, 22 (12), pp 1913–1921<br>• J CHEMINFORMATICS, 2016 , 8 (1) :6

Property	Predicted values	Probability	Suggestions	Meaning & Preference	Reference
DILI (Drug Induced Liver Injury)	+	0.538		• The DILI positive(+) & negative(-) classification criteria refers the reference.	• J CHEM INF MODEL, 2015, 55(10) :2085-2093

VASP-05

Physicochemical Property

Property	Predicted values	Suggestions	Meaning & Preference	Reference
LogS (Solubility)	-5.367 log mol/L (1.893 μg/mL)	> 10 μg/ml	• Optimal: higher than -4 log mol/L<br>• <10 μg/mL: Low solubility; 10–60 μg/mL: Moderate solubility; >60 μg/mL: High solubility	• Book: ISBN: 9787562832287. pp. 14<br>• J PHARMACOL TOX MET. 2000, 44 (1), 235−249;
$LogD_{7.4}$ (Distribution Coefficient D)	2.782	1~5	• < 1: Solubility high; Permeability low by passive transcellular diffusion; Permeability possible via paracellular if MW < 200; Metabolism low.<br>• 1 to 3: Solubility moderate; Permeability moderate; Metabolism low.<br>• 3 to 5: Solubility low; Permeability high; Metabolism moderate to high.<br>• > 5: Solubility low; Permeability high; Metabolism high.	• Methods and principles in medicinal chemistry 18 (pp. 21–45). Weinheim: Wiley-VCH.
LogP (Distribution Coefficient P)	4.884	0~3	• Optimal: 0< LogP <3<br>• LogP <0: poor lipid bilayer permeability.<br>• LogP >3: poor aqueous solubility.	• Book: ISBN: 3-906390-22-5. pp. 127–182.

Absorption

Property	Predicted values	Probability	Suggestions	Meaning & Preference	Reference
Papp (Caco-2 Permeability)	-4.731 cm/s		> -5.15 cm/s	Optimal: higher than -5.15 Log unit or -4.70 or -4.80	• J CHEM INF MODEL. 2016, 56 (4), pp 763–773.
Pgp-inhibitor	+	0.616		• The Pgp-inhibitor & non-inhibitor classification criteria refers the reference.	• J CHEM INF MODEL. 2010. 50(6): p. 1034-1041.<br>• J MED CHEM. 2011. 54(6): p. 1740-1751.
Pgp-substrate	---	0.014		• More likely to be a Pgp substrate: N+O ≥ 8; MW > 400; Acid with pKa > 4<br>• More likely to be a Pgp non-substrate: N+O ≤ 4; MW < 400; Acid with pKa < 8	• J DRUG TARGET. 11, 391–406.
HIA (Human Intestinal Absorption)	++	0.779		• ≥30%: HIA+; <30%: HIA-	• RSC ADV. 2017, 7, 19007-19018

Property	Predicted values	Probability	Suggestions	Meaning & Preference	Reference
F (20% Bioavailability)	+	0.631		• ≥20%: F20+; <20%: F20-	• MOL PHARMACEUT, 2011. 8(3): p. 841-851<br>• J PHARMACEUT BIOMED, 2008. 47(4): p. 677-682.
F (30% Bioavailability)	+	0.587		• ≥30%: F30+; <30%: F30-	• MOL PHARMACEUT, 2011. 8(3): p. 841-851<br>• J PHARMACEUT BIOMED, 2008. 47(4): p. 677-682.

Distribution

Property	Predicted values	Probability	Suggestions	Meaning & Preference	Reference
PPB (Plasma Protein Binding)	91.064 %		90%	• Significant with drugs that are highly protein-bound and have a low therapeutic index.	• ISBN: 978-0-1236-9520-8. pp. 194
VD (Volume Distribution)	0.065 L/kg		0.04~20 L/kg	• Optimal: 0.04-20L/kg;<br>• Range: <0.07L/kg: Confined to blood, Bound to plasma protein or highly hydrophilic; 0.07-0.7L/kg: Evenly distributed; >0.7L/kg: Bound to tissue components (e.g., protein, lipid),highly lipophilic.	• Book: ISBN: 9787562832287. pp. 174<br>• Book: ISBN: 978-0-1236-9520-8. pp. 229
BBB (Blood–Brain Barrier)	++	0.822		• BB ratio >=0.1: BBB+; BB ratio <0.1: BBB-<br>• These features tend to improve BBB permeation: H-bonds (total) < 8–10; MW < 400–500; No acids.	• J NEUROCHEM. 70, 1781–1792

Metabolism

Property	Predicted values	Probability	Meaning & Preference	Reference
P450 CYP1A2 inhibitor	+	0.528	• Molecules that labeled inhibitor in PubChem BioAssay were regarded as inhibitor.	• NAT BIOTECHNOL. 2009, 27(11): 1050-1055.<br>• BIOINFORMATICS. 2013, 29(16): 2051-2052.

Property	Predicted values	Probability	Meaning & Preference	Reference
P450 CYP1A2 Substrate	+	0.621	• Molecules that labeled substrate in PubChem BioAssay were regarded as substrate.<br>• Characteristics of CYP1A2 substrate: 0.08< LogP <3.61; Planar amines and amides	• NAT BIOTECHNOL. 2009, 27(11): 1050-1055.<br>• BIOINFORMATICS. 2013, 29(16): 2051-2052.
P450 CYP3A4 inhibitor	-	0.493	• Molecules that labeled inhibitor in PubChem BioAssay were regarded as inhibitor.<br>• Strategies to Reduce CYP3A4 Inhibition: Decrease the lipophilicity (LogD $_{7.4}$); Add steric hindrance to the heterocycle para to the nitrogen; Add an electronic substitution (e.g., halogen) that reduces the pKa of the nitrogen.	• NAT BIOTECHNOL. 2009, 27(11): 1050-1055.<br>• BIOINFORMATICS. 2013, 29(16): 2051-2052.
P450 CYP3A4 substrate	-	0.498	• Molecules that labeled substrate in PubChem BioAssay were regarded as substrate.<br>• Characteristics of CYP3A4 substrate: 0.97< LogP <7.54; Large molecules	• NAT BIOTECHNOL. 2009, 27(11): 1050-1055.<br>• BIOINFORMATICS. 2013, 29(16): 2051-2052.<br>• ISBN: 978-0-1236-9520-8. pp. 162
P450 CYP2C9 inhibitor	-	0.313	• Molecules that labeled inhibitor in PubChem BioAssay were regarded as inhibitor.	• NAT BIOTECHNOL. 2009, 27(11): 1050-1055.<br>• BIOINFORMATICS. 2013, 29(16): 2051-2052.
P450 CYP2C9 substrate	+	0.517	• Molecules that labeled substrate in PubChem BioAssay were regarded as substrate.<br>• Characteristics of CYP2C9 substrate: 0.89< LogP <5.18; Acidic (Nonionized)	• MOL INFORM. 2011. 30(10): p. 885-895.<br>• J CHEM INF MODEL. 2013. 53(12): p. 3373-3383.<br>• ISBN: 978-0-1236-9520-8. pp. 162
P450 CYP2C19 inhibitor	++	0.822	• Molecules that labeled inhibitor in PubChem BioAssay were regarded as inhibitor.	• NAT BIOTECHNOL. 2009, 27(11): 1050-1055.<br>• BIOINFORMATICS. 2013, 29(16): 2051-2052.

Property	Predicted values	Probability	Meaning & Preference	Reference
P450 CYP2C19 substrate	-	0.431	• Molecules that labeled substrate in PubChem BioAssay were regarded as substrate.	• NAT BIOTECHNOL. 2009, 27(11): 1050-1055.<br>• BIOINFORMATICS. 2013, 29(16): 2051-2052.
P450 CYP2D6 inhibitor	-	0.385	• Molecules that labeled inhibitor in PubChem BioAssay were regarded as inhibitor.	• MOL INFORM. 2011. 30(10): p. 885-895.<br>• J CHEM INF MODEL. 2013. 53(12): p. 3373-3383.
P450 CYP2D6 substrate	-	0.32	• Molecules that labeled substrate in PubChem BioAssay were regarded as substrate.<br>• Characteristics of CYP2D6 substrate: 0.75< LogP <5.04; Basic (Ionized)	• MOL INFORM. 2011. 30(10): p. 885-895.<br>• J CHEM INF MODEL. 2013. 53(12): p. 3373-3383.<br>• ISBN: 978-0-1236-9520-8. pp. 162

Elimination

Property	Predicted values	Suggestions	Meaning & Preference	Reference
$T_{1/2}$ (Half Life Time)	1.781 h	> 0.5 h	• Range: >8h: high; 3h< Cl < 8h: moderate; <3h: low	• ISBN: 978-0-1236-9520-8. pp. 236
CL (Clearance Rate)	1.156 mL/min/kg		• Range: >15 mL/min/kg: high; 5mL/min/kg< Cl < 15mL/min/kg: moderate; <5 mL/min/kg: low	• ISBN: 978-0-1236-9520-8. pp. 236

Toxicity

Property	Predicted values	Probability	Suggestions	Meaning & Preference	Reference
hERG (hERG Blockers)	+++	0.94		• Where molecules with IC50 < 40 μM were regarded as blockers.<br>• Features may lead to hERG blocker:<br>o A basic amine (positively ionizable, pKa >7.3).<br>o Hydrophobic/lipophilic substructure(s) (ClogP >3.7).<br>o Absence of negatively ionizable groups or oxygen H-bond acceptors.	• TRENDS PHARMACOL SCI. 2005, 26(3): 119-124<br>• ISBN: 978-0-1236-9520-8. pp. 213<br>• MOL PHARM. 2016, 13(8):2855–2866
H-HT (Human Hepatotoxicity)	++	0.756		• The H-HT positive(+) & negative(-) classification criteria refers the reference.	• CHEM RES TOXICOL, 2016, 29(5): 757-767.
AMES (Ames Mutagenicity)	-	0.32		• Ames positive(+) & negative(-): significantly induces revertant colony growth at least in one out of usually five strains, otherwise, negative.	• J CHEM INF MODEL. 2012, 52(11): 2840-2847.
SkinSen (Skin sensitization)	-	0.458		• Sensitizer & Non-sensitizer: The (r)LLNA experimental value. (r)LLNA: (Reduced) local lymph node assay.	• TOXICOL APPL PHARM, 2015 , 284 (2) :262-272
LD50 (LD50 of acute toxicity)	2.439 -log mol/kg (1604.09 mg/kg)		> 500 mg/kg	• Median lethal dose (LD50) usually represents the acute toxicity of chemicals.It is the dose amount of a tested molecule to kill 50 % of the treated animals within a given period.<br>• High-toxicity: 1~50 mg/kg; Toxicity: 51~500 mg/kg; low-toxicity: 501~5000 mg/kg.	• CHEM RES TOXICOL, 2009, 22 (12), pp 1913–1921<br>• J CHEMINFORMATICS, 2016 , 8 (1) :6

Property	Predicted values	Probability	Suggestions	Meaning & Preference	Reference
DILI (Drug Induced Liver Injury)	+	0.538		• The DILI positive(+) & negative(-) classification criteria refers the reference.	• J CHEM INF MODEL, 2015, 55(10) :2085-2093

VASP-06

Physicochemical Property

Property	Predicted values	Suggestions	Meaning & Preference	Reference
LogS (Solubility)	-5.387 log mol/L (1.808 μg/mL)	> 10 μg/ml	• Optimal: higher than -4 log mol/L<br>• <10 μg/mL: Low solubility; 10–60 μg/mL: Moderate solubility; >60 μg/mL: High solubility	• Book: ISBN: 9787562832287. pp. 14<br>• J PHARMACOL TOX MET. 2000, 44 (1), 235−249;
$LogD_{7.4}$ (Distribution Coefficient D)	2.796	1~5	• < 1: Solubility high; Permeability low by passive transcellular diffusion; Permeability possible via paracellular if MW < 200; Metabolism low.<br>• 1 to 3: Solubility moderate; Permeability moderate; Metabolism low.<br>• 3 to 5: Solubility low; Permeability high; Metabolism moderate to high.<br>• > 5: Solubility low; Permeability high; Metabolism high.	• Methods and principles in medicinal chemistry 18 (pp. 21–45). Weinheim: Wiley-VCH.
LogP (Distribution Coefficient P)	4.884	0~3	• Optimal: 0< LogP <3<br>• LogP <0: poor lipid bilayer permeability.<br>• LogP >3: poor aqueous solubility.	• Book: ISBN: 3-906390-22-5. pp. 127–182.

Absorption

Property	Predicted values	Probability	Suggestions	Meaning & Preference	Reference
Papp (Caco-2 Permeability)	-4.672 cm/s		> -5.15 cm/s	Optimal: higher than -5.15 Log unit or -4.70 or -4.80	• J CHEM INF MODEL. 2016, 56 (4), pp 763–773.
Pgp-inhibitor	+	0.63		• The Pgp-inhibitor & non-inhibitor classification criteria refers the reference.	• J CHEM INF MODEL. 2010. 50(6): p. 1034-1041.<br>• J MED CHEM. 2011. 54(6): p. 1740-1751.
Pgp-substrate	---	0.061		• More likely to be a Pgp substrate: N+O ≥ 8; MW > 400; Acid with pKa > 4<br>• More likely to be a Pgp non-substrate: N+O ≤ 4; MW < 400; Acid with pKa < 8	• J DRUG TARGET. 11, 391–406.
HIA (Human Intestinal Absorption)	++	0.779		• ≥30%: HIA+; <30%: HIA-	• RSC ADV. 2017, 7, 19007-19018

Property	Predicted values	Probability	Suggestions	Meaning & Preference	Reference
F (20% Bioavailability)	+	0.631		• ≥20%: F20+; <20%: F20-	• MOL PHARMACEUT, 2011. 8(3): p. 841-851<br>• J PHARMACEUT BIOMED, 2008. 47(4): p. 677-682.
F (30% Bioavailability)	+	0.536		• ≥30%: F30+; <30%: F30-	• MOL PHARMACEUT, 2011. 8(3): p. 841-851<br>• J PHARMACEUT BIOMED, 2008. 47(4): p. 677-682.

Distribution

Property	Predicted values	Probability	Suggestions	Meaning & Preference	Reference
PPB (Plasma Protein Binding)	91.183 %		90%	• Significant with drugs that are highly protein-bound and have a low therapeutic index.	• ISBN: 978-0-1236-9520-8. pp. 194
VD (Volume Distribution)	0.127 L/kg		0.04~20 L/kg	• Optimal: 0.04-20L/kg;<br>• Range: <0.07L/kg: Confined to blood, Bound to plasma protein or highly hydrophilic; 0.07-0.7L/kg: Evenly distributed; >0.7L/kg: Bound to tissue components (e.g., protein, lipid),highly lipophilic.	• Book: ISBN: 9787562832287. pp. 174<br>• Book: ISBN: 978-0-1236-9520-8. pp. 229
BBB (Blood–Brain Barrier)	++	0.808		• BB ratio >=0.1: BBB+; BB ratio <0.1: BBB-<br>• These features tend to improve BBB permeation: H-bonds (total) < 8–10; MW < 400–500; No acids.	• J NEUROCHEM. 70, 1781–1792

Metabolism

Property	Predicted values	Probability	Meaning & Preference	Reference
P450 CYP1A2 inhibitor	+	0.519	• Molecules that labeled inhibitor in PubChem BioAssay were regarded as inhibitor.	• NAT BIOTECHNOL. 2009, 27(11): 1050-1055.<br>• BIOINFORMATICS. 2013, 29(16): 2051-2052.

Property	Predicted values	Probability	Meaning & Preference	Reference
P450 CYP1A2 Substrate	+	0.557	• Molecules that labeled substrate in PubChem BioAssay were regarded as substrate.<br>• Characteristics of CYP1A2 substrate: 0.08< LogP <3.61; Planar amines and amides	• NAT BIOTECHNOL. 2009, 27(11): 1050-1055.<br>• BIOINFORMATICS. 2013, 29(16): 2051-2052.
P450 CYP3A4 inhibitor	++	0.742	• Molecules that labeled inhibitor in PubChem BioAssay were regarded as inhibitor.<br>• Strategies to Reduce CYP3A4 Inhibition: Decrease the lipophilicity (LogD $_{7.4}$); Add steric hindrance to the heterocycle para to the nitrogen; Add an electronic substitution (e.g., halogen) that reduces the pKa of the nitrogen.	• NAT BIOTECHNOL. 2009, 27(11): 1050-1055.<br>• BIOINFORMATICS. 2013, 29(16): 2051-2052.
P450 CYP3A4 substrate	-	0.444	• Molecules that labeled substrate in PubChem BioAssay were regarded as substrate.<br>• Characteristics of CYP3A4 substrate: 0.97< LogP <7.54; Large molecules	• NAT BIOTECHNOL. 2009, 27(11): 1050-1055.<br>• BIOINFORMATICS. 2013, 29(16): 2051-2052.<br>• ISBN: 978-0-1236-9520-8. pp. 162
P450 CYP2C9 inhibitor	-	0.491	• Molecules that labeled inhibitor in PubChem BioAssay were regarded as inhibitor.	• NAT BIOTECHNOL. 2009, 27(11): 1050-1055.<br>• BIOINFORMATICS. 2013, 29(16): 2051-2052.
P450 CYP2C9 substrate	-	0.368	• Molecules that labeled substrate in PubChem BioAssay were regarded as substrate.<br>• Characteristics of CYP2C9 substrate: 0.89< LogP <5.18; Acidic (Nonionized)	• MOL INFORM. 2011. 30(10): p. 885-895.<br>• J CHEM INF MODEL. 2013. 53(12): p. 3373-3383.<br>• ISBN: 978-0-1236-9520-8. pp. 162
P450 CYP2C19 inhibitor	++	0.776	• Molecules that labeled inhibitor in PubChem BioAssay were regarded as inhibitor.	• NAT BIOTECHNOL. 2009, 27(11): 1050-1055.<br>• BIOINFORMATICS. 2013, 29(16): 2051-2052.

Property	Predicted values	Probability	Meaning & Preference	Reference
P450 CYP2C19 substrate	-	0.397	• Molecules that labeled substrate in PubChem BioAssay were regarded as substrate.	• NAT BIOTECHNOL. 2009, 27(11): 1050-1055.<br>• BIOINFORMATICS. 2013, 29(16): 2051-2052.
P450 CYP2D6 inhibitor	-	0.406	• Molecules that labeled inhibitor in PubChem BioAssay were regarded as inhibitor.	• MOL INFORM. 2011. 30(10): p. 885-895.<br>• J CHEM INF MODEL. 2013. 53(12): p. 3373-3383.
P450 CYP2D6 substrate	-	0.307	• Molecules that labeled substrate in PubChem BioAssay were regarded as substrate.<br>• Characteristics of CYP2D6 substrate: 0.75< LogP <5.04; Basic (Ionized)	• MOL INFORM. 2011. 30(10): p. 885-895.<br>• J CHEM INF MODEL. 2013. 53(12): p. 3373-3383.<br>• ISBN: 978-0-1236-9520-8. pp. 162

Elimination

Property	Predicted values	Suggestions	Meaning & Preference	Reference
$T_{1/2}$ (Half Life Time)	1.852 h	> 0.5 h	• Range: >8h: high; 3h< Cl < 8h: moderate; <3h: low	• ISBN: 978-0-1236-9520-8. pp. 236
CL (Clearance Rate)	0.982 mL/min/kg		• Range: >15 mL/min/kg: high; 5mL/min/kg< Cl < 15mL/min/kg: moderate; <5 mL/min/kg: low	• ISBN: 978-0-1236-9520-8. pp. 236

Toxicity

Property	Predicted values	Probability	Suggestions	Meaning & Preference	Reference
hERG (hERG Blockers)	+++	0.936		• Where molecules with IC50 < 40 μM were regarded as blockers.<br>• Features may lead to hERG blocker:<br>○ A basic amine (positively ionizable, pKa >7.3).<br>○ Hydrophobic/lipophilic substructure(s) (ClogP >3.7).<br>○ Absence of negatively ionizable groups or oxygen H-bond acceptors.	• TRENDS PHARMACOL SCI. 2005, 26(3): 119-124<br>• ISBN: 978-0-1236-9520-8. pp. 213<br>• MOL PHARM. 2016, 13(8):2855–2866
H-HT (Human Hepatotoxicity)	++	0.716		• The H-HT positive(+) & negative(-) classification criteria refers the reference.	• CHEM RES TOXICOL, 2016, 29(5): 757-767.
AMES (Ames Mutagenicity)	-	0.32		• Ames positive(+) & negative(-): significantly induces revertant colony growth at least in one out of usually five strains, otherwise, negative.	• J CHEM INF MODEL. 2012, 52(11): 2840-2847.
SkinSen (Skin sensitization)	-	0.458		• Sensitizer & Non-sensitizer: The (r)LLNA experimental value. (r)LLNA: (Reduced) local lymph node assay.	• TOXICOL APPL PHARM, 2015 , 284 (2) :262-272
LD50 (LD50 of acute toxicity)	2.446 -log mol/kg (1578.443 mg/kg)		> 500 mg/kg	• Median lethal dose (LD50) usually represents the acute toxicity of chemicals.It is the dose amount of a tested molecule to kill 50 % of the treated animals within a given period.<br>• High-toxicity: 1~50 mg/kg; Toxicity: 51~500 mg/kg; low-toxicity: 501~5000 mg/kg.	• CHEM RES TOXICOL, 2009, 22 (12), pp 1913–1921<br>• J CHEMINFORMATICS, 2016 , 8 (1) :6

Property	Predicted values	Probability	Suggestions	Meaning & Preference	Reference
DILI (Drug Induced Liver Injury)	+	0.538		• The DILI positive(+) & negative(-) classification criteria refers the reference.	• J CHEM INF MODEL, 2015, 55(10) :2085-2093
